# *BRCA1/*2-negative, high-risk breast cancers (*BRCAX*) for Asian women: genetic susceptibility loci and their potential impacts

**DOI:** 10.1038/s41598-018-31859-8

**Published:** 2018-10-15

**Authors:** Joo-Yeon Lee, Jisun Kim, Sung-Won Kim, Sue K. Park, Sei Hyun Ahn, Min Hyuk Lee, Young Jin Suh, Dong-Young Noh, Byung Ho Son, Young Up Cho, Sae Byul Lee, Jong Won Lee, John L. Hopper, Joohon Sung

**Affiliations:** 1 Department of Health Science, Graduate School of Public Health, Seoul National University, Seoul, Republic of Korea; 2 Department of Surgery, University of Ulsan College of Medicine, Asan Medical Center, Seoul, Republic of Korea; 3 Department of Surgery, Daerim St. Mary’s Hospital, Seoul, Republic of Korea; 4 Department of Preventive Medicine, Seoul National University College of Medicine, Seoul, Republic of Korea; 5 Department of Biomedical Science, Seoul National University College of Medicine, Seoul, Republic of Korea; 6 Cancer Research Institute, Seoul National University College of Medicine, Seoul, Republic of Korea; 7 Department of Surgery, Soonchunhyang University Seoul Hospital, Seoul, Republic of Korea; 8 Department of Surgery, St. Vincent’s Hospital, The Catholic University of Korea School of Medicine, Seoul, Republic of Korea; 9 Department of Surgery, Seoul National University College of Medicine, Seoul, Republic of Korea; 10 Department of Surgery, Yonsei University College of Medicine, Yonsei Cancer Center, Seoul, Republic of Korea; 11 Centre for Epidemiology and Biostatistics, University of Melbourne, Carlton, Victoria, Australia; 12 Institute of Health & Environment, Seoul National University, Seoul, Republic of Korea; 13 Bio-MAX Institute, Seoul National University, Seoul, Republic of Korea

## Abstract

*“BRCAX”* refers breast cancers occurring in women with a family history predictive of being a *BRCA1*/2 mutation carrier, but *BRCA1/2* genetic screening has failed to find causal mutations. In this study, we report the findings of the genetic architecture of *BRCAX* with novel and redefined candidate loci and their potential impacts on preventive strategy. We performed a genome-wide association study involving 1,469 *BRCAX* cases from the Korean Hereditary Breast Cancer study, and high-risk breast cancer cases (1,482 Asians and 9,902 Europeans) from the Breast Cancer Association Consortium. We also evaluated the previously reported susceptibility loci for their roles in the high-risk breast cancers. We have identified three novel loci (*PDE7B*, *UBL3*, and a new independent marker in *CDKN2B-AS1*) associated with *BRCAX*, and replicated previously reported SNPs (24 of 92) and moderate/high-penetrance (seven of 23) genes for Korean *BRCAX*. For the novel candidate loci, evidence supported their roles in regulatory function. We estimated that the common low-penetrance loci might explain a substantial part of high-risk breast cancer (39.4% for Koreans and 24.0% for Europeans). Our study findings suggest that common genetic markers with lower penetrance constitute a part of susceptibility to high-risk breast cancers, with potential implications for a more comprehensive genetic screening test.

## Introduction

Breast cancer is the most common female cancer worldwide, and its incidence rate is increasing rapidly in Asian populations including South Korea^1,2^. The National Cancer Screening Program in Korea provides mammography tests for screening breast cancer for women aged 40–69 years^3^. The effectiveness of this screening program, however, is controversial mainly because the validity of mammography-based test is lower for Asian women than for Caucasians^4–8^ About 4.8% and 11.1% of breast cancers were diagnosed before age 40 and 45, respectively, in the United States between 2005 and 2014. The proportions of these early-onset cases almost tripled for Korean women during the same period (13.9% and 29.8%), although overall incidence rate is less than a half of that for Western women^9,10^. Early-onset breast cancer cases are generally more likely to have higher pathologic grade and to be receptor-negative tumors^11–14^. Given the low predictive value of mammography for Asian women, ultrasonographic breast examination might be introduced to improve the validity but at greater cost. An effective genetic screening test, if available, might be able to identify women at high-risk of breast cancers for personalized prevention programs. So far, to our knowledge, the genetic etiology of high-risk breast cancers is understudied particularly for Asian women with more burdens from high-risk breast cancers.

Mutations in *BRCA1* and *BRCA2* (hereafter referred to as *BRCA1/2*), the most important genetic susceptibility markers, account for up to 15% and 30% of the familial recurrence risk (FRR) of overall and high-risk breast cancers^15^. Clinical practice guidelines are well-established for *BRCA1/2* mutation carriers^16^. Conversely, lack of proper knowledge has left the *BRCA1/2* mutation non-carriers’ genetic counseling largely unattended, although these non-carriers with strong family history show similar clinical presentations predictive of *BRCA1/2* mutation carriers (“*BRCAX*” cases), with four-fold or greater lifetime risk of breast cancer than the general population^17,18^. In Korea, breast cancer cases with early-onset (age < 40) or other indications suggesting a strong genetic burden are entitled to *BRCA1/2* mutation testing covered by Korean National Health Insurance. The test has found clinically significant *BRCA1/2* mutations for only 15.7% of eligible patients (22.3% of examinees indicated by strong family history, and 8.8% by early-onset)^19^, to make the understanding of “*BRCAX* genes” a priority. Several studies have been conducted to elucidate the genetic architecture of *BRCAX* cancers^20–22^. Studies do not have replicated the novel loci from each other, suggesting that susceptibility loci for *BRCAX* might be population-specific, at least partly. Asian women might have specific genetic susceptibility for the high-risk breast cancers, but, to our knowledge, no studies have been conducted toward this end.

We conducted a genome-wide search for *BRCAX* genes involving 1,469 Korean female breast cancer cases who were eligible for the *BRCA1/2* test but negative for causal mutations. Then, we replicated our findings using the Breast Cancer Association Consortium (BCAC) data. We also estimated the role of previously reported susceptibility loci on the risk of *BRCAX* occurrence. Finally, we evaluated the impacts and estimated risk of those associated markers on high-risk breast cancers for Asians and Europeans.

## Results

For the discovery phase, a total of 1,478 Korean *BRCAX* cases and 5,979 controls were selected with 3,378,933 markers (flowchart is shown in Supplementary Fig. S1). Characteristics of the participants were described in Table 1. Mean age of cases (40.2) was approximately 15 years younger compared to controls (55.1), but allele frequencies of associated markers were not significantly different between age group (Supplementary Table S1). Among 1,469 cases after quality control, 169 and 288 women had one *BRCA1* or *BRCA2* mutation with unverified significance. For *BRCA2* gene, we found 42 variants significantly which are more frequent in the cases compared to the controls (Supplementary Table S2). However, the ClinVar database showed that 40 of these were reported to be benign and only two variants (rs2126042 and rs766173) was reported to be benign or have uncertain significance.

**Table 1:** Characteristics of breast cancer cases (n = 1,469).

Variables		No. (%)
Age (years old), mean ± sd^a^		40.24 ± 9.13
*BRCA1/2* mutation^b^	*BRCA1*–neg & *BRCA2*–uv	288 (19.6%)
	*BRCA1*–uv & *BRCA2*–neg	169 (11.5%)
	Both negative	1012 (68.9%)
Age of breast cancer onset	≤40	1037 (70.6%)
	40–45	143 (9.7%)
	>45	289 (19.7%)
Family history of breast cancer in the first or second degree relatives	no history	908 (61.8%)
	1	492 (38.2%)
	2	60 (4.1%)
	3	9 (0.6%)
Family history of ovarian cancer in the first or second degree relatives	no history	1399 (95.2%)
	1	68 (4.6%)
	2	2 (0.1%)
Disease history of cancers other than breast cancer		72 (4.9%)
Bilateral breast cancer		122 (8.3%)
Receptor status		
Estrogen receptor	negative	465 (31.7%)
	positive	891 (60.7%)
	unknown	113 (7.7%)
Progesterone receptor	negative	496 (33.8%)
	positive	879 (59.8%)
	unknown	94 (6.4%)
HER2 receptor	negative	924 (62.9%)
	positive	275 (18.7%)
	equivocal^c^	139 (9.5%)
	unknown	131 (8.9%)
Triple-negative		223 (15.2%)

^a^Sd: standard deviation.

^b^Neg: negative, uv: unverified mutation.

^c^Immunohistochemistry (IHC) 2+, Fluorescence *In Situ* Hybridization (FISH) unknown.

Being indicated by early-onset (≤age 40) comprised the largest proportion of the case composite (70.6%), followed by having a family history of breast cancer (42.9%).

**Figure 1:**
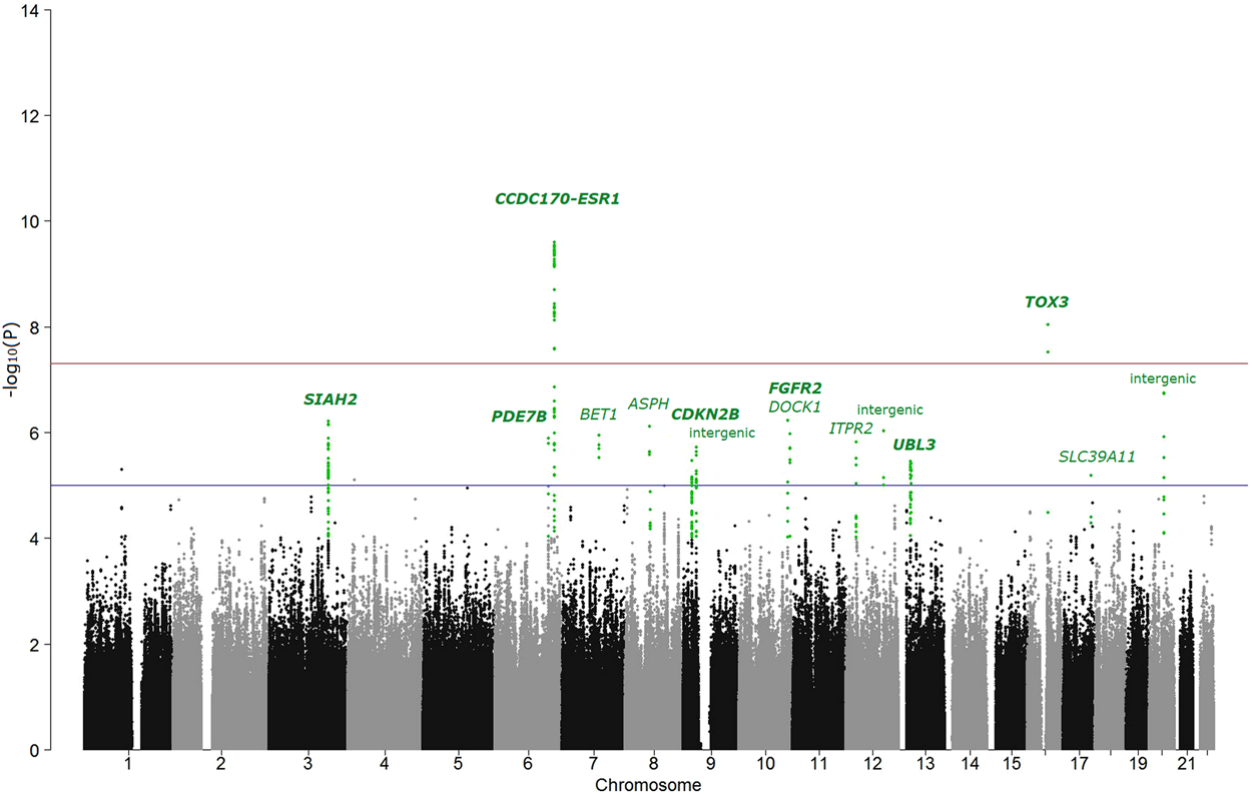
Manhattan plot. The red horizontal line represents the genome-wide significance threshold of *p*-value = 5.0 × 10^−8^ and the blue horizontal line represents the suggestive significance threshold of *p*-value = 1.0 × 10^−5^. For significantly associated regions, SNPs with *p*-value less than 10^-3^ are highlighted in green and the replicated genes are marked in bold.

From the genome-wide association study (GWAS), the genomic inflation factor (ʎ_GC_) was 1.09 and *p*-values were corrected for the inflation factor. Figure 1 and Supplementary Fig. S2 show the Manhattan plot and the quantile-quantile plot. Initially, 15 independent loci of single nucleotide polymorphisms (SNPs) showed *p*-values < 10^−5^. Two SNPs reached genome-wide significance level in the first phase analysis, and both were reported before: rs9383936 (*p* = 1.01 × 10^−10^, OR = 1.35) on the intron of *CCDC170* (near *ESR1*) and rs4784227 (*p* = 9.11 × 10^−9^, OR = 1.31) on the intron of *TOX3*. Three previously reported loci (*SIAH2*, *FGFR2*, *PGAM1P5/NTN4*) and nine new loci also comprised the initial 15 loci. One variant on *CDKN2B-AS1* (rs1011970) was previously associated with breast cancer risk for European descendants^23,24^, but our study found another variant in the proximal regions of the *CDKN2B-AS1* gene, rs78545330. These two SNPs constituted separate haplotype blocks (r^2^ = 0.026 for East Asians), and we considered rs78545330 as independent.

The replication set consisted of 1,482 cases (1,194 early-onset cases and/or 390 women with a family history breast cancer in first-degree relatives, Asian high-risk cases) and 3,612 control women without family history of breast cancer. We compared the characteristics and composite of high-risk breast cancer cases of different populations (Supplementary Table S3). Among the initial 15 candidate loci, six variants were statistically significant (at *p*-value < 0.05) including two previously known (*CCDC170/ESR1*, and *TOX3*) and four novel susceptibility loci. Among four novel loci, rs11154838, located on 6q23.3 (intron of *PDE7B*), showed associations at genome-wide significance level (*p*-values: 2.27 × 10^−8^). Other two novel candidate loci (rs78545330 in the intron of *CDKN2B-AS1* and rs278050 on 13q12.3, 73 Kb 5′ of *UBL3*), while replicated in Asian high-risk cases at nominal *p*-value, did not reach genome-wide significance in the meta-analysis (*p*-values: 8.56 × 10^−8^ and 1.63 × 10^−6^). The other novel variant, rs4969001 on *SLC39A11*, was associated in the opposite direction; thus, it was not considered for further analysis. We summarize findings of these 15 candidate loci in Table 2. For European high-risk breast cancers, all three novel susceptibility loci did not show meaningful associations (*p*-values = 0.34, 0.24 and 0.50).

**Table 2:** Genomic loci associated with breast cancer (*P*-value < 10^−5^).

SNP	Chr	Position (hg19)	Nearest Genes (distance to gene)	Function	Risk Allele (fwd)	RAF in 1 kG Phase3^a^		Korean *BRCAX* cases (initial)			Asian high-risk cases (replication)			Meta-analysis		
						EAS	EUR	RAF in cases^a^	*P*-value	OR (95% CI)^b^	RAF in cases^a^	*P*-value	OR (95% CI)^b^	*P*-value	OR (95% CI)^b^	*P*_hetero_^c^
rs60538652	3	150473606	*SIAH2*	Intron	A	0.61	0.98	0.65	6.24.E-07	1.26 (1.15–1.38)	0.73	0.072	1.13 (0.99–1.28)	3.25.E-07	1.21 (1.13–1.31)	0.16
rs11154838	6	136290942	*PDE7B*	Intron	C	0.72	0.65	0.75	1.28.E-06	1.27 (1.15–1.40)	0.83	0.005	1.26 (1.07–1.49)	2.27.E-08	1.27 (1.17–1.38)	0.96
rs9383936	6	151944614	3′ of *CCDC170* (2.3 Kb), 5′ of *ESR1* (3.3 Kb)	Intergenic	A	0.31	0.08	0.34	1.01.E-10	1.35 (1.23–1.48)	0.34	1.05.E-06	1.3 (1.17–1.44)	6.35.E-16	1.33 (1.24–1.42)	0.58
rs10953105	7	93622247	*BET1*	3′ UTR	G	0.86	0.97	0.88	1.13.E-06	1.37 (1.21–1.56)	0.91	0.192	1.14 (0.94–1.38)	1.81.E-06	1.30 (1.16–1.44)	0.11
rs2350923	8	62605259	*ASPH*	Intron	T	0.72	0.70	0.76	7.75.E-07	1.28 (1.16–1.42)	0.27	0.750	1.02 (0.92–1.13)	0.250	1.14 (0.91–1.44)	0.00
rs78545330	9	21995941	*CDKN2B-AS1*	Intron	A	0.21	0.08	0.26	3.41.E-06	1.26 (1.14–1.39)	0.20	5.73.E-03	1.19 (1.05–1.35)	8.56.E-08	1.23 (1.14–1.33)	0.47
rs10814070	9	34129839	3′ of *DCAF12* (3.1 Kb)	Intergenic	T	0.71	0.25	0.75	1.93.E-06	1.26 (1.15–1.39)	0.65	0.469	0.96 (0.87–1.07)	0.465	1.10 (0.85–1.44)	0.00
rs2912774	10	123348662	*FGFR2*	Intron	T	0.38	0.43	0.42	5.96.E-07	1.25 (1.14–1.36)	0.43	0.052	1.11 (1.00–1.21)	5.60.E-07	1.18 (1.10–1.25)	0.06
rs9418690	10	128809949	*DOCK1*	Intron	C	0.73	0.76	0.76	1.08.E-06	1.28 (1.16–1.41)	0.82	0.819	1.02 (0.85–1.23)	0.183	1.16 (0.93–1.44)	0.04
rs4964006	12	26770889	*ITPR2*	Intron	T	0.95	0.69	0.98	1.52.E-06	1.96 (1.49–2.58)	0.95	0.804	0.97 (0.76–1.24)	0.366	1.38 (0.69–2.75)	0.00
rs67129489	12	96016957	5′ of *PGAM1P5* (26.0 Kb), 5′ of *NTN4* (34.6 Kb)	Intergenic	G	0.13	0.32	0.15	9.31.E-07	1.36 (1.20–1.53)	0.06	0.976	1.00 (0.77–1.32)	0.226	1.20 (0.89–1.60)	0.05
rs278050	13	30436968	5′ of *UBL3* (73 Kb)	Intergenic	C	0.19	0.23	0.21	3.89.E-06	1.28 (1.15–1.42)	0.19	0.050	1.13 (1.00–1.28)	1.64.E-06	1.22 (1.12–1.32)	0.14
rs4784227	16	52583143	*TOX3*	Intron	T	0.26	0.25	0.32	9.11.E-09	1.31 (1.19–1.44)	0.29	6.24.E-05	1.25 (1.12–1.39)	3.26.E-12	1.28 (1.20–1.38)	0.49
rs4969001	17	70980127	*SLC39A11*	Intron	T	0.89	0.96	0.86	6.53.E-06	1.32 (1.17–1.49)	0.89	0.041	0.85 (0.73–0.99)	0.780	1.06 (0.69–1.64)	0.00
rs73107564	20	36263775	—	Intergenic	A	0.89	0.81	0.89	1.81.E-07	1.43 (1.25–1.63)	0.87	0.541	0.96 (0.83–1.10)	0.432	1.17 (0.79–1.73)	0.00

^a^RAF: risk allele frequency, EAS: East Asian, EUR: European.

^b^OR: odds ratio, CI: confidence interval.

^c^*P*_hetero_: *P*-value for heterogeneity. Fixed effect model was used if p-value for heterogeneity exceeds 0.05; otherwise random effect model was used.

When we conducted a meta-analysis by the receptor-status, four loci of the initial 15 candidate loci from the GWAS showed receptor-status specific associations with even smaller sample sizes. We only focused on findings where smaller subgroup resulted in the stronger association, to reduce the influence of sample size variation. rs60538652, on *SIAH2*, rs2912774 on *FGFR2* and rs67129489 near *PGAM1P5/NTN4* were more strongly associated with ER+ or PR+ cases, while rs9383936 near *ESR1* was more strongly associated with ER− or PR− breast cancers (*P*_*heterogeneity*_ <0.05). It is noteworthy that markers at *SIAH2*, *FGFR2*, and *PGAM1P5/NTN4* showed an increased level of significance in subgroup analyses despite smaller sample sizes. None of the variants identified from the GWAS were differentially associated according to HER2 receptor status or being TNBC. More details about the receptor-status-wise analyses were described in Supplementary Table S4.

Three novel candidate regions were visualized using LD plots and regional association plots (Supplementary Fig. S3) and annotated for regulatory elements (Supplementary Table S5). Two markers (rs6905776 and rs12174235), in the same LD block with rs11154838 on *PDE7B*, were annotated for regulatory elements (GWAS *p*-values: 1.63 × 10^−6^ and 2.72 × 10^−5^). rs78545330 on the intron of *CDKN2B-AS1* and correlated markers showed strong evidence of regulatory function from multiple tissues including breast tissues, while rs1011970 (previously reported SNP) was not annotated for regulatory elements. East Asians have a risk allele of rs78545330 (A, allele frequency = 0.21) three times more frequently than Europeans (0.08). We presented the haplotype structures of this region between Asian and Europeans (Supplementary Fig. S4). For East Asians, multiple LD blocks were more distinct, and the LD between rs78545330 and rs1011970 was weak in East Asians (r^2^ = 0.02), indicating that rs78545330 is a novel candidate variant. When we examined the genetic regions harboring candidate variant near *UBL3*, the region consisted of two independent LD blocks, and rs278050 and correlated SNPs showed enhancer-related histone marks in several tissues including breasts and mammary tissues. Also, rs73444211 in the other LD block near *UBL3* participated in diverse regulatory functions.

Results from eQTL analyses for three candidate loci also showed evidence of associations with gene-expression levels of different tissues (Supplementary Table S6). The risk allele T of rs11154838 was associated with *PDE7B* gene-expression level in blood vessels (p-value = 0.001), but not with other tissues. rs78545330 on *CDKN2B* showed strong associations with gene expressions in multiple tissues. The risk allele substantially decreases the expression of *CDKN2B*, a tumor suppressor gene (*p*-value = 1.7 × 10^−13^, effect size = −0.44). rs278050 near *UBL3* also altered cis-gene regulation in several tissues.

When we replicated findings from the previous studies, a part (24 for Korean *BRCAX*, 55 for European and 14 for Asian high-risk breast cancers) of 92 loci from the GWAS catalog showed meaningful associations (*p*-value < 0.05 with the same direction of association as in the previous reports, Supplementary Table S7). Generally, replicated variants showed larger risk for *BRCAX* or high-risk cancers than the previous reports for all breast cancers. Since the majority of known susceptibility markers were reported from studies of European populations, the number of replicated markers was larger for European cases compared with Korean or Asian cases. However, the sum of %FRR (Σ%FRR) of each replicated marker was largest for Korean *BRCAX* cases (29.8%), followed by European high-risk cases (24.0%) and Asian high-risk cases (10.6%). Three novel candidate variants accounted for additional 9.6% and 3.0% FRR for Koreans and Asians. It is noteworthy that three replicated variants on *FGFR2*, *TOX3*, and *ESR1* have been reported in previous searches for *BRCAX* genes^20–22^. These variants were also associated with overall breast cancer risk in multiple studies^25^. Other candidate loci reported from previous *BRCAX* studies were not replicated in this study^20–22^. Finally, among the breast cancer predisposition genes with moderate/high-penetrance^16,26^, seven genes (*BMPR1A*, *PTEN*, *CDH1*, *NF1*, *RAD51C*, *BRIP1*, and *CHEK2*) were replicated for Korean *BRCAX* (eight for Asian and 15 genes for European high-risk breast cancers, Supplementary Table S8). The estimated contributions from the moderate/high-penetrance variants varied by the populations, accounting for 4.2%, 13.2%, and 7.9% of overall high-risk breast cancers for Koreans, Asians, and Europeans.

**Figure 2:**
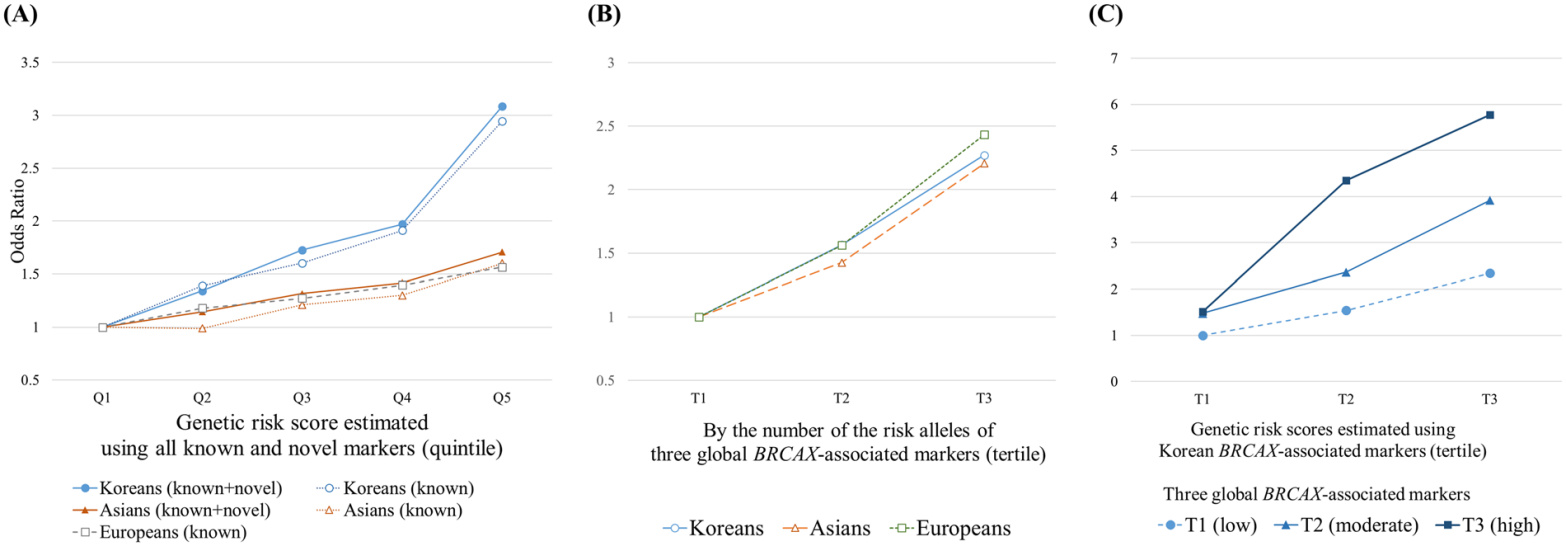
Predicted risks by genetic risk scores. (**A**) Relative risks by genetic risk scores (GRSs) across the population. For two different types of markers sets (“*BRCAX*-known”: markers replicated for Korean *BRCAX* cases among previously reported breast cancer-associated markers from the GWAS catalog; “*BRCAX*-known + novel”: three novel candidate markers added to replicated markers), a predictive performance by GRSs was estimated for Korean *BRCAX*, Asian, and European high-risk cases. For *BRCAX*-known markers, predictive performance by GRSs was highest for Korean *BRCAX* followed by European and Asian high-risk cases. When three novel markers were added, however, prediction improved for Asians compared to Europeans. (**B**) Predicted risks of breast cancer according to three risk groups using the number of risk alleles of three consistently reported markers from three previous GWAS of *BRCAX* across the population: *FGFR2* (rs2912774), *TOX3* (rs4784227), and *ESR1* (rs9383936). (**C**) Predicted *BRCAX* risks by GRSs using *BRCAX*-known markers by tertile groups of three global *BRCAX*-associated markers (on *FGFR2*, *TOX3*, and near *ESR1*): circle symbols are for low-risk group (T1), triangle symbols are for moderate risk group (T2), and square symbols are for high-risk group (T3).

We estimated the increase in risk with increase in genetic risk score (GRS) for each population (Fig. 2A). Predicted risk (OR) by GRS was highest for Korean *BRCAX* cases, and the OR increased upon adding newly identified markers for both Koreans (OR: from 2.95 to 3.09) and Asians (OR: from 1.60 to 1.71). Also, we estimated the genetic risk of *BRCAX* according to genotypes of “shared between populations (*FGFR2/TOX3/ESR1*, reported from multiple *BRCAX* studies including ours)”. The estimated increases in the risk of *BRCAX* or high-risk breast cancers from the variants on *FGFR2*, *TOX3*, and *ESR1* were quite similar across populations (ORs for highest compared to lowest tertile = 2.2 to 2.4, Fig. 2B). When we stratified and compared the estimated risk of GRSs calculated from the three novel and 21 replicated (after exclusion of three markers on *FGFR2/TOX3/ESR1* among 24 replicated variants) “Asian-specific *BRCAX”* markers according to “shared between population” (*FGFR2/TOX3/ESR1*) genotype status, those with multiple risk alleles of *FGFR2/TOX3/ESR1* showed steeper increases in risk for breast cancers, compared with those in none or one allele groups (Fig. 2C).

## Discussion

From a genome-wide search, three candidate loci were newly identified with suggestive evidence of functional involvement. The three novel loci identified by this study might tag the closely-located functional variants based on the downstream analyses. The marker itself, or markers within the LD block, have marks for regulatory elements, suggesting these SNPs themselves or variants tagged by the SNPs might be associated with the pathophysiology of *BRCAX* breast cancer development. *PDE7B* (phosphodiesterase 7B) encodes a cyclic adenosine monophosphate (cAMP)-specific phosphodiesterase and *CDKN2B* (cyclin-dependent kinase 4 inhibitor B) is a known tumor suppressor gene^27^. The risk allele of the top hit (rs78545330) on *CDKN2B-AS1* showed strong evidence of reducing gene expression level of this tumor suppressor gene.

When we examined the association of newly identified markers with overall Asian cases including late-onset and sporadic breast cancers of BCAC, both rs11154838 (*PDE7B*) and rs78545330 (*CDKN2B-AS1*) showed stronger level of significance but with smaller effect sizes (ORs: 1.16 and 1.14 for all breast cancers, 1.26 and 1.19 for high-risk breast cancers), but rs278050 (*UBL3*) was not associated (*p* = 0.2). For European cases, rs78545330 was replicated for overall cases only (p = 0.017, OR = 1.04; high-risk breast cancer: p = 0.2), but other two novel candidate loci did not show significant associations. One possible explanation for this population-specific manner of association is that genetic susceptibility to *BRCAX* might comprise multiple genetic loci which are only partly shared between populations. The newly identified candidate loci might be Asian-specific and also more associated with the early-onset type of high-risk cancers. When we estimated OR according to GRS, the addition of novel variants to known markers increased the risk for Korean and Asian, but not for European high-risk breast cancers.

It is interesting that some of the markers shared between high-risk and general breast cancers showed augmented impacts on the *BRCAX* cancers. For example, rs12628403 on the intron of *APOBEC3A* alone explained 6.5% of FRR (OR = 1.49), compared with OR = 1.17 for overall breast cancers. Also, rs10069690 on the intron of *TERT* was replicated for *BRCAX* with higher risk estimate (OR = 1.24) than the OR (1.10) from the original studies. These findings are in line with the findings from the colorectal cancer study^28^: some SNPs show stronger associations with high-risk cases with larger impacts. Our findings suggest that role of some variants might not be same between in high-risk and in general breast cancers, although they are associated with both types. It might be logical to infer that a substantial part of the genetic risk of high-risk breast cancers comprises common variants overlapping with general types of breast cancers but at augmented risks.

Among replicated markers, *FGFR2* (rs2912774), *TOX3* (rs4784227), and *ESR1* (rs9383936) deserve a special remark. All three previous GWAS for *BRCAX* replicated *FGFR2*, and multiple studies replicated *TOX3* and *ESR1*^20–22^. Our study also replicated these three variants for high-risk breast cancers in all population data. When we compared the estimated risk of these three variants, the estimated risk was quite similar between three populations (Korean *BRCAX*, Asian and European high-risk cancer, Fig. 2B). This finding suggests that shared genetic susceptibility toward high-risk breast cancer might exist across the population. These three variants might be only the tip of the iceberg. Our study did not examine sequence level variation which might also be shared across populations. At least, our findings support that a part of common variants associated with low-risk breast cancers also has their roles in the susceptibility of high-risk breast cancer.

In our study, about 62% of Korean *BRCAX* cases, 48% and 25% of Asian and European high-risk cancers were non-familial early-onset cases. Europeans have more familial cancers probably due to higher prevalence than Asians or inclusion criteria of each study. It is not clear whether two major indications of being a “high-risk” type, i.e., early-onset and familial cases have differences in their genetic constitutions. In our study, differences in the association and effect size between populations for some variants might stem from the differences in this case composites.

When GRS was re-estimated according to the risk allele counts of *FGFR2*/*TOX3*/*ESR1*, the estimated risk from GRS not only was larger but also showed a steeper increase suggesting an effect modification. The risk modification according to *FGFR2*/*TOX3*/*ESR1* was more evident for *BRCAX* (Korean), partly because of its strict case definitions (Fig. 2C).

We also evaluated the impact of known and novel susceptibility loci for high-risk breast cancers. The estimated Σ%FRR from replicated markers was 29.8% for Korean *BRCAX* with 24 markers. The findings from replicated markers (29.8% FRR) might not be attributed to so-called “winner’s curse” bias, an inflated impact estimation in the discovery set itself, because the Korean *BRCAX* served as a replication set. Three novel loci accounted for 9.6% of the FRR for Korean *BRCAX*. The impact of three novel loci on Korean *BRCAX* cases, however, might be inflated for having used the discovery set on itself. Novel candidate loci added 3.0% to Asian, but none to European high-risk cancers. When the %FRR of three novel loci was estimated from all types of Asian breast cancers, the estimate decreased to 0.75%, suggesting that these loci mainly account for the genetic risk of high-risk breast cancers.

Sum of FRR (Σ%FRR) from common variants are considerably compatible between populations: 29.8–39.4% for Koreans, and 24.0% for Europeans, and 10.6–13.6% for other Asians. One reason why Σ%FRR is smaller for Asians might be explained their heterogenic ethnic compositions (In this study, Asian high-risk breast cancer cases consisted of Japanese 28%, Chinese 30%, Asian American 16%, and others 25%). If associated markers have not been identical between ethnic groups, some variants more specifically associated with certain populations might be missed. Moreover, replication was conducted using 92 markers, most of them were reported from studies of European ancestry. Also, a smaller sample size could be another reason for fewer replicated markers in Asians. Wen *et al*.’s study replicated 44 of 78 previously reported loci involving 11,760 overall breast cancer cases of East Asians^29^. When we performed an analysis including late-onset breast cancer cases (6,130 cases and 6,371 controls), we could have replicated 24 loci (10 consistent, 12 added and two removed loci). Whether the difference is attributable to the power or the differences in the genetic susceptibility between high-risk and late-onset breast cancers is not conclusive. The six additional loci, however, did not show any clues of possible association with high-risk breast cancers (p > 0.3), and we believe the differences in the genetic architecture between sporadic/low-risk and familial/high-risk breast cancers might explain a part of the discrepancy. Korean data might have shown compatible Σ%FRR of high-risk breast cancers with Europeans, partly due to their size and stricter definition for cases. We believe it might be reasonable to interpret the findings that common variants explain as much Σ%FRR of high-risk cancers as *BRCA1/2* mutations. We also examined the associations and their impacts on breast cancer predisposition genes with moderate/high-penetrance. Although imputed markers covered most of the known variants, our approach might have materially underestimated their impacts, and cannot replace the findings from the sequence-based approach.

A proper genetic screening program for *BRCA/BRCAX*, if implemented, might contribute to detecting high-risk women for providing personalized screening program such as ultrasonography. Additionally, it will provide information for consulting the relatives of *BRCAX* cases. Findings from our study support the necessity and possibility of developing a new genetic screening for *BRCAX*. However, our study alone does not provide other important information necessary for effective genetic screening, in particular, estimates of penetrance and predictive values of the test. Estimating sensitivity and specificity of *BRCAX*-associated markers are required first in larger data. Also, this study considered the genetic markers associated with the occurrence of high-risk breast cancer, and their potential implications for detecting high-risk populations. To provide evidence that support a new genetic screening program, further data about a reduced mortality rate from breast cancer might be required, but these are beyond scope of ours.

We believe that this study confers some advantages over previous *BRCAX* studies. First, the number of test-proven *BRCA*-negative cases was not modest (n = 1,469), and more than 11,300 high-risk breast cancer cases were included, larger than previous studies^20–22^. Second, all the *BRCA1*/*2* mutation status was more accurately identified by standard sequencing analysis of each *BRCA1* and *BRCA2* exon. Third, we selected high-risk cases with genetic burdens using the similar indications as *BRCA1/2* mutation test: age of onset, family history, history of other malignancy, and bilateral cancers. Applying these strict indications might have enabled us to select cases with high genetic burdens. Fourth, we attempted to re-evaluate the findings from our own GWAS study and previous studies jointly including both common variants in the GWAS catalog, and moderate/high-penetrance genes. This approach might have provided more comprehensive views on the genetic architecture of *BRCAX* and high-risk breast cancers. Similarly, this approach enabled us to estimate the impacts of associated markers on the occurrence of *BRCAX* for different populations and context. The impact estimates from our study will confer implications for genetic screening test of high-risk women. Finally, to our knowledge, this is the first Asian study focused on *BRCAX* cases, where epidemiologic features suggest unique susceptibility might exist.

This study also has several limitations. First, exact information about *BRCA1/2* mutation status was scarce, and not available for most studies of the BCAC. Applying the criteria for high-risk cancers only which were not confirmed to be *BRCA1/2*-negative might have decreased the power of detecting associated markers. Nevertheless, this limitation might not have influenced toward increasing false-positive findings in the study. Second, Asian cases in the BCAC data were limited so that the criteria for early-onset age were relaxed to the age 45, while the same criteria for having family history was applied. These relaxed criteria in the replication data might have reduced the power to detect susceptibility loci more specific to the *BRCAX* cases. When we estimated the associations for replicated and novel loci using different age criteria for Asian high-risk cases (938 cases for replication set when age 40 was used), direction and strength of associations were largely same with slightly less statistical power: the number of replicated markers and Σ%FRR of them declined from 14 (10.6%) to eight (9.0%). However, ORs of three novel markers increased and Σ%FRR increased from 3.0% to 3.8% accordingly. Third, even with the sample size of 1,469 confirmed *BRCAX* cases, our study has limitations in power to newly detect relatively rare alleles (0.01 < MAF < 0.05) because of the scarcity of high-risk cancer cases. We estimated that the relative risk should exceed 2.5 to be detected as novel with our sample size^30^. Fourth, although this not being the main purpose of our study, when we attempted to replicate the “moderate/high-penetrance” variants, the effects might have been underestimated because some of the variants were not exactly identified in our imputed genotype data. We believe the estimated impacts from these genes should have been underestimated. The estimated impact of the common variants, however, is not likely affected by this limitations, because the frequency of the common risk alleles is independent of the rare alleles. Fifth, the replication analysis of previously reported loci might have inflated as the replication set could include data of the discovery set. Finally, as the mean age was different by affection status in the discovery set (cases: 40.2, controls: 55.1), the age was not used as a covariate, because we did not observe any difference in allele frequencies between controls (older) and those in the population with same age with cases (younger) (Supplementary Table S1). If there are some susceptibility loci which are expressed in an age-dependent manner, those markers could not have considered in this study. However, we believe older controls might be more appropriate in part due to they have lower chance of developing breast cancer compared to younger controls with similar mean age with the cases (40 years old).

In conclusion, we have identified novel common susceptibility loci from GWAS and replicated some of both common/low-penetrance and rare/high-penetrance variants which were reported previously. We also evaluated the contribution of those susceptibility loci including ours, to high-risk breast cancers of multiple populations. Our findings also suggest that high-risk breast cancers, particularly for Asians, might consist of multiple layers with similar importance, including *BRCA1/2*, moderate/high-penetration genes, and selected common variants with augmented effects. Our study might add information to consider prevention strategy of early-onset and high-risk breast cancers in Asia, and also to the genetic counseling for relatives of the high-risk breast cancer patients without a mutation in *BRCA1/2* genes.

## Methods

### Study design and participants of the discovery set

We conducted a case-control GWAS. Breast cancer patients were selected from the Korean Hereditary Breast Cancer (KOHBRA) study. Breast cancer patients with one of following five conditions and family members of *BRCA1/2* mutation carriers were eligible for *BRCA1/2* gene test provided by the Korean National Health Insurance - family history of breast or ovarian cancer, diagnosed at age 40 or younger, bilateral breast cancer, male cases, or diagnosed with another primary malignancy. The KOHBRA study enrolled those who provided informed consent among the “*BRCA1/2* gene test eligible” cases and their family members. The detailed information of the KOHBRA study has been described previously^31^ and in Supplementary Notes. From the KOHBRA participants, unrelated women (estimated identity by descent < 0.125) who met the following criteria were included in this study:

(i) Breast cancer patients who were tested to be free of clinically significant *BRCA1/2* gene mutation (clinically unverified mutation cases were included)
  AND at least one of the following
(ii) Diagnosed as breast cancer before or at age 40; OR having one or more relative(s) with breast/ovarian cancer; OR bilateral cases; OR previously diagnosed as other cancer.

We used data from Korean Genome Epidemiology Study (KoGES) as control. The profiles of KoGES consortium was described elsewhere^32^ and in Supplementary Notes. Genetically unrelated (estimated identity by descent <0.125) Korean women without family history of breast or ovarian cancer were included. All participants gave informed written/oral consent, and this study was approved by the Institutional Review Board of Asan Medical Center, Seoul, Republic of Korea. This study was conducted in accordance with the World Medical Association Declaration of Helsinki.

### Genotyping and imputation methods

Cases were genotyped using Illumina OncoArray-500K Beadchip at the University of Southern California Epigenome Center, CA, USA. Peripheral blood samples (20 ml) were collected at baseline, and they were divided, treated and stored within 24 hours of sampling. We excluded individuals with low call rate (<95%) and SNPs with genotype call rate <95%, minor allele frequency (MAF)<1%, evidence for deviation from Hardy-Weinberg equilibrium at *p*-value < 10^−9^, or poor cluster plots. Genotype data were phased and imputed using SHAPEIT and IMPUTE2 software respectively, and East Asian data from the 1000 Genomes Project (1 kG) Phase 3 were used as reference^33–36^. From imputed markers, SNPs with info score greater than 0.95 were included. We genotyped cases and controls with four different platforms of dense genome-wide SNP arrays and conducted standard quality control and imputation process. And the batch effect detected through association analysis of control data genotyped from different platforms was removed (*p* < 0.01). For post-imputation quality control, markers with high missing rate >10%, minor allele frequency (MAF)<1%, evidence for deviation from Hardy-Weinberg equilibrium at *p*-value < 10^−6^ were excluded. More detailed information was described in Supplementary Notes.

### BRCA1 and BRCA2 genomic mutation analysis

Genomic DNA was extracted from the participants’ peripheral blood samples. For *BRCA1/2* mutation testing in cases, 22 coding exons of *BRCA1* and 26 coding exons of *BRCA2* were scanned through fluorescence-based conformation sensitive gel electrophoresis (F-CSGE) and denaturing high-performance liquid chromatography (DHPLC). For a subset of PCR products with aberrant patterns, direct sequencing was performed on an ABI3100 or ABI3700 (Applied Biosystems, CA) or a MegaBACE500 (GE Healthcare, UK) genetic analyzer. In this study, the definition of a genetic mutation is restricted to the protein-truncating mutation and the missense mutation, which are known to be associated with the disease. This study also includes the participants with a clinically unverified mutation in *BRCA1/2* genes. More detailed information about *BRCA1/2* mutation analysis has been described before^31,37^. Since this study also includes the cases with unverified mutation on *BRCA1/2*, we examined whether non-pathogenic variants on these genes are more frequent in the cases compared to controls at p-value of 0.05. And we searched them at the ClinVar database.

### Statistical analyses for the genome-wide search

GWAS was carried out using logistic regression by trends test of PLINK version 1.07^38^. For visualization of the results, Manhattan plot and quantile-quantile (QQ) plot were generated using R package ‘qqman’^39^. Principal component analysis (PCA) was conducted using EIGENSTRAT, and the first three principal components were used for adjustment^40^. Since most cases were early-onset breast cancer and cohorts for control recruited adults with a limited age range, mean age was much younger in cases than controls. Thus, allele frequencies of significant markers were presented by affection status and age groups, rather than adjusting for age.

### Replication and meta-analysis of newly identified loci

For the replication of the initial candidate loci, imputed data of Asian population from the BCAC were used. The overall description about the BCAC was previously reported^41^ and in Supplementary Notes. Since most of the cases in the BCAC lack *BRCA1/2* mutation information, we included high-risk breast cancers defined as: breast cancer cases diagnosed before or at age 45 or cases with family history of breast cancer in first degree relatives. For controls, women without family history of breast cancer were included among the BCAC control data.

For newly identified loci, we conducted meta-analyses using fixed or random effect model according to evidence of heterogeneity (fixed effect model was used if *p*-value for heterogeneity exceeds 0.05). Also, we conducted subgroup analyses by three receptor status (estrogen, progesterone, and HER2 receptor). *P*-values for heterogeneity by receptor status were calculated using a Cochran’s Q test, and a *p*-value of 0.05 was used to determine heterogeneity.

### Downstream analyses of newly identified loci

For newly identified loci which were replicated using Asian high-risk cases from the BCAC, downstream analyses were performed. These regions were visualized by linkage disequilibrium (LD) plots and regional association plots. LD plots were created using control data with ‘LDheatmap’ package in R^42^. And regional association plots were created by Locuszoom using East Asian population of the 1 kG Phase 3 as reference^43^.

Functional information of novel loci was annotated using Encyclopedia of DNA elements (ENCODE) project and HaploReg version 4.1^44,45^. Novel SNPs and correlated markers with r^2^ > 0.8 from East Asian population were explored. Results of expression quantitative trait loci (eQTL) analyses were found from the Genotype Tissue Expression (GTEx) portal^46^. Associations between novel markers and expression of the nearest gene of each SNP for all available tissues and associations between these markers and nearby genes with a window size of 1 Mb for the tissue showing the most significant associations were retrieved.

### Replicating susceptibility loci previously associated with breast cancers

We estimated associations between previously reported breast cancer susceptibility loci and *BRCAX* cases and how the significance of associations and their effect sizes differ. First, we selected markers associated with breast cancer at p-value < 10^−5^ from the NHGRI-EBI GWAS catalog^25^. We also examined the candidate loci from three previous *BRCA1/2*-negative cancer GWAS^20–22^. Finally, the variants on genes associated with increased risk of breast or ovarian cancer listed in the guideline for genetic screening of high-risk breast cancers (suggested by the National Comprehensive Cancer Network, NCCN) or breast cancer predisposition genes used in a recent article were evaluated in this study^16,26^. Seventeen genes listed on the NCCN guideline were *ATM*, *CDH1*, *CHEK2*, *MSH2*, *MLH1*, *MSH6*, *PMS2*, *EPCAM*, *NBN*, *NF1*, *PALB2*, *PTEN*, *RAD51C*, *RAD51D*, *STK11*, and *TP53*, except for *BRCA1/2*; six additional genes suggested from the study by Wong *et al*. were *BARD1*, *BMPR1A*, *FANCC*, *SMAD4*, *VHL*, and *XRCC2*. Variants in gene regions ± 10 kB were tested for association. In this analysis, the markers with MAF < 1%, which were excluded during quality control, were also included. Independent (r^2^ > 0.3) SNPs with *p*-value < 0.01 were included for impact estimation.

To assess the impact of reported loci in the occurrence of *BRCAX* cases, we estimated the FRRs from Korean *BRCAX* women, Asian and European high-risk women using the BCAC data (cases with age of onset <45 or with a family history of breast cancer in first-degree relatives, for both Asian and European data). The fraction of FRR (%FRR) explained by markers was calculated as ln(λ)/ln(λ_0_) where lambda (λ) is the FRR to a relative of an affected individual and λ_0_ is the overall FRR. λ is calculated as (pr^2^ × q)/(pr + q)^2^ where p is the frequency of the risk allele in the general population, q equals 1 − p, and r is the per-allele OR. And λ_0_ is assumed to be 1.8 as reported from the previous article^47,48^. For comparison of FRR explained by each SNP, we applied the same allele frequencies from the East Asian or European data from the 1 kG Phase 3 and different odds ratios (ORs) from each analysis.

### Impact analysis for novel and replicated loci

To compare predictive power among different groups, we estimated ORs according to GRSs. GRSs were calculated as a weighted sum of the number of risk alleles, in which the weight was taken as the natural log of the OR from association analyses. ORs by quintiles of GRS were estimated using the first quintile as the reference. We compared the predictive power of *BRCAX* by the different set of markers for different populations (Korean *BRCAX*, Asian and European high-risk cases).

We re-evaluated the ORs from polygenic GRS, by stratifying subjects according to their risk alleles associated with *BRCAX* in multiple studies^20–22^. GRSs were calculated using markers replicated for Korean *BRCAX* among previously reported markers in breast cancer from the GWAS catalog (“*BRCAX*-known”), and with novel candidate markers from the GWAS added to replicated markers (“*BRCAX*-known + novel”).

## Electronic supplementary material

Supplementary Materials

## Data Availability

The datasets generated and/or analysed during the current study are available in the Open Science Framework repository, https://osf.io/a7kn5/. The data that support the findings of this study are available from BCAC but restrictions apply to the availability of these data, which were used under license for the current study, and so are not publicly available. Data are however available from the authors upon reasonable request and with permission of the BCAC group.
