# Supplementary material for: BRCA1/2-negative, high-risk breast cancers (BRCAX) for Asian women: genetic susceptibility loci and their potential impacts
Source: Sci Rep. 2018 Oct 15;8:15263. doi: 10.1038/s41598-018-31859-8 (PMC6189145; doi:10.1038/s41598-018-31859-8)
Supplement: Supplementary file 1 — Supplementary Materials [file 41598_2018_31859_MOESM1_ESM.docx]

**SUPPLEMENTARY INFORMATION FOR THE MANUSCRIPT ENTITLED:**

***BRCA1/2*-negative, high-risk breast cancers (*BRCAX*) for Asian women: genetic susceptibility loci and their potential impacts**

Joo-Yeon Lee^1*^, Jisun Kim^2*^, Sung-Won Kim^3^, Sue K. Park ^4,5^, Sei Hyun Ahn^2^, Min Hyuk Lee^6^, Young Jin Suh^7^, Dong-Young Noh^5,8^, Byung Ho Son^2^, Young Up Cho^9^, Sae Byul Lee^2^, Jong Won Lee^2**^, John L. Hopper^1,10**^ and Joohon Sung^1, 11,12**^

^1^ Department of Health Science, Graduate School of Public Health, Seoul National University, Seoul, Republic of Korea

^2^ Department of Surgery, University of Ulsan College of Medicine, Asan Medical Center, Seoul, Republic of Korea

^3^ Department of Surgery, Daerim St. Mary’s Hospital, Seoul, Republic of Korea

^4^ Department of Preventive Medicine, Seoul National University College of Medicine, Seoul, Republic of Korea; Department of Biomedical Science, Seoul National University College of Medicine, Seoul, Republic of Korea

^5^ Cancer Research Institute, Seoul National University College of Medicine, Seoul, Republic of Korea

^6^ Department of Surgery, Soonchunhyang University Seoul Hospital, Seoul, Republic of Korea

^7^ Department of Surgery, St. Vincent’s Hospital, The Catholic University of Korea School of Medicine, Seoul, Republic of Korea

^8^ Department of Surgery, Seoul National University College of Medicine, Seoul, Republic of Korea

^9^ Department of Surgery, Yonsei University College of Medicine, Yonsei Cancer Center, Seoul, Republic of Korea

^10^ Centre for Epidemiology and Biostatistics, University of Melbourne, Carlton, Victoria, Australia

^11^ Institute of Health & Environment, Seoul National University, Seoul, Republic of Korea

^12^ Bio-MAX Institute, Seoul National University, Seoul, Republic of Korea

^*^ These authors contributed equally to this work.

^**^ These authors jointly supervised this work.

**Supplementary Notes (Participating Studies)**

***Korean Hereditary Breast Cancer (KOHBRA) study***

All cases were selected from KOHBRA study. KOHBRA study is a prospective multicenter cohort with the aim to estimate the prevalence of *BRCA1/2* mutations and ovarian cancer from high-risk patients of hereditary breast cancer and their families. Breast cancer patients with one of following five conditions and family members of *BRCA1/2* mutation carriers were eligible for *BRCA1/2* gene test provided by the Korean National Health Insurance - family history of breast or ovarian cancer, diagnosed at age 40 or younger, bilateral breast cancer, male cases, or diagnosed with another primary malignancy. The KOHBRA study enrolled those who provided informed consent among the “*BRCA1/2* gene test eligible” cases and their family members. From October 2007 to May 2013, it enrolled 3,143 subjects and breast cancer cases without pathogenic mutation on *BRCA1* or *BRCA2* were included for this analysis. Participants included for this study were genotyped using Illumina OncoArray-500K Beadchip. More detailed information was described before ^1^.

***Korean Genome and Epidemiology Study (KoGES)***

All controls were selected from studies of KoGES. KoGES is a consortium project consisting of six prospective cohort studies aiming to establish a genome epidemiological study platform and to investigate the genetic and environmental etiology of common complex diseases for Koreans and causes of death with long-term follow-up. Quality control and imputation of genotype data were conducted using the same criteria applied in the process of genotypes data of cases. More information can be found from the previous articles ^2-4^ and selected cohorts for this study were described as follows.

1. *The Healthy Twin Study*

The Healthy Twin study is a cohort of healthy adult same-sex twins and their family members, aged 17-81 years. This study aimed to elucidate genetic and environmental factors underlying complex diseases and traits. A total of 3,500 participants were enrolled from 2005 to 2015, and 3,447 individuals were genotyped on either Affymetrix Genome-Wide Human SNP Array 6.0 or Illumina HumanCore-24 Beadchip.

1. *Health Examinee (HEXA) shared control study*

The HEXA cohort was initiated in 2001 aiming to identify risk factors of life-style related complex diseases such as type 2 diabetes, hypertension, and dyslipidemia. Subjects were recruited from the national health examinee registry, aged 40-69 years. From over 173k individuals, 4,302 subjects were genotyped with the Affymetrix Genome-Wide Human SNP array 6.0.

1. *Health2 study*

Health2 study is another community-based cohort of KoGES consortium. A total of 8,500 individuals, aged 40-69 years, were recruited from five rural areas of Korea. Genotyping was performed using Affymetrix Genome-Wide Human SNP array 6.0.

1. *Cardiovascular disease association study (CAVAS) cohort*

CAVAS is population-based cohort of KoGES consortium. Participants were recruited from among residents of three rural cities of Korea. A total of 8,702 individuals, aged 40-69 years participated and 4,052 healthy participants with no history of hypertension, type 2 diabetes, hyperlipidemia, heart disease, blood vessel disease of the brain, or cancer were selected for SNP genotyping. Genotyping was conducted using Illumina HumanOmni1-Quad Beadchip.

***Breast Cancer Association Consortium (BCAC)***

Data from BCAC was used for replication analysis of novel variants and impact analysis of all known and novel loci. Samples of both Asian and European ancestry were included. More detailed information was described elsewhere ^5^.

For replication and meta-analysis of novel candidate loci, seven studies of Asian breast cancer were included: SBCGS (Shanghai Breast Cancer Genetic Study, China), HERPACC (Hospital-based Epidemiologic Research Program at Aichi Cancer Center, Japan), TWBCS (Taiwanese Breast Cancer Study), ACP (Asia Cancer Program, Thailand), and MYBRCA (Malaysian Breast Cancer Genetic Study). SEBCS (Seoul Breast Cancer Study, Korea) and LAABC (Los Angeles County Asian-American Breast Cancer Case-Control Study) were not included, as there was only one or no control women without family history of breast cancer (family history is unknown for majority of control women). For analysis of high-risk cases, only cases with early onset (diagnosed before age 45) or with family history of breast cancer and controls without family history of breast cancer were included. For analysis of all cases, all breast cancer cases and all control women were included regardless of age of onset or family history. SEBCS and LAABC were also included.

For European high-risk cases, the same inclusion criteria as used for Asian high-risk cases were applied and following 19 studies were included. KCONFAB/AOCS (Kathleen Cuningham Foundation Consortium for research into Familial Breast Cancer/Australian Ovarian Cancer Study, Australia), HMBCS (Hannover-Minsk Breast Cancer Study, Belarus), MARIE, Mammary Carcinoma Risk Factor Investigation, Germany), CECILE (CECILE Breast Cancer Study, France), MCBCS (Mayo Clinic Breast Cancer Study, USA), ESTHER (ESTHER Breast Cancer Study, Germany), SBCS (Sheffield Breast Cancer Study, UK), GENICA (Gene Environment Interaction and Breast Cancer in Germany, Germany) SEARCH (Study of Epidemiology and Risk factors in Cancer Heredity, UK), PBCS (NCI Polish Breast Cancer Study, Poland), OFBCR (Ontario Familial Breast Cancer Registry, Canada), UKBGS (UK Breakthrough Generations Study, UK), BBCS (British Breast Cancer Study, UK), KBCP (Kuopio Breast Cancer Project, Finland), ABCS (Amsterdam Breast Cancer Study, Netherlands), SZBCS (IHCC-Szczecin Breast Cancer Study, Poland), ABCFS (Australian Breast Cancer Family Study, Australia), CTS (California Teachers Study, USA) and NBCS (Norwegian Breast Cancer Study, Norway).

For both data, logistic regression analyses were conducted using PLINK v1.07. For Asian samples, age, the first two principal components and study sites were used for adjustment. For Europeans, age, the first six principal components and study sites were used for adjustment.

Supplementary Table S1. Allele frequencies by different age groups in Korean control population.

| **SNP** | **Position (hg19)** | **Risk Allele** | **RAF^a^ in Cases  (n=1469)** | **RAF^a^ in controls**  **by age groups** | | | **P-value for trend** |
| --- | --- | --- | --- | --- | --- | --- | --- |
|  |  |  |  | **<45 (n=988)** | **45-59 (n=2809)** | **≥60 (n=2182)** |  |
| rs60538652 | 3:150473606 | A | 0.65 | 0.60 | 0.60 | 0.59 | 0.9978 |
| rs11154838 | 6:136290942 | C | 0.75 | 0.70 | 0.70 | 0.70 | 0.9938 |
| rs9383936 | 6:151944614 | A | 0.34 | 0.29 | 0.27 | 0.27 | 0.9982 |
| rs10953105 | 7:93622247 | G | 0.88 | 0.83 | 0.83 | 0.85 | 0.9912 |
| rs2350923 | 8:62605259 | T | 0.76 | 0.71 | 0.71 | 0.71 | 0.9955 |
| rs78545330 | 9:21995941 | A | 0.26 | 0.20 | 0.22 | 0.22 | 0.9982 |
| rs10814070 | 9:34129839 | T | 0.75 | 0.70 | 0.70 | 0.70 | 0.9717 |
| rs2912774 | 10:123348662 | T | 0.42 | 0.38 | 0.37 | 0.36 | 0.9981 |
| rs9418690 | 10:128809949 | C | 0.76 | 0.71 | 0.71 | 0.71 | 0.9976 |
| rs4964006 | 12:26770889 | T | 0.98 | 0.96 | 0.96 | 0.96 | 0.9765 |
| rs67129489 | 12:96016957 | G | 0.15 | 0.11 | 0.12 | 0.11 | 0.9890 |
| rs278050 | 13:30436968 | C | 0.21 | 0.18 | 0.17 | 0.18 | 0.9860 |
| rs4784227 | 16:52583143 | T | 0.32 | 0.27 | 0.27 | 0.26 | 0.9988 |
| rs4969001 | 17:70980127 | T | 0.86 | 0.83 | 0.82 | 0.82 | 0.9948 |
| rs73107564 | 20:36263775 | A | 0.89 | 0.85 | 0.85 | 0.85 | 0.9730 |

^a^ RAF, risk allele frequency

Supplementary Table S2. Variants more frequent in the cases compared to controls (on *BRCA2*)

| ***CHR*** | ***SNP*** | ***BP*** | ***Minor***  ***Allele*** | ***MAF***  ***in cases*** | ***MAF***  ***in controls*** | ***P*** | ***OR*** |
| --- | --- | --- | --- | --- | --- | --- | --- |
| *13* | *rs11571591* | *32894738* | *A* | *0.1328* | *0.1193* | *0.03384* | *1.13* |
| *13* | *rs11571597* | *32896676* | *A* | *0.1429* | *0.1298* | *0.04721* | *1.118* |
| *13* | *rs11571598* | *32896722* | *T* | *0.1429* | *0.1298* | *0.04721* | *1.118* |
| *13* | *rs11571605* | *32898238* | *A* | *0.1431* | *0.1298* | *0.04314* | *1.12* |
| *13* | *rs11571610* | *32899388* | *C* | *0.1431* | *0.1298* | *0.04364* | *1.12* |
| *13* | *rs11571618* | *32899838* | *T* | *0.1431* | *0.13* | *0.04579* | *1.118* |
| *13* | *rs3783265* | *32900149* | *C* | *0.1431* | *0.1298* | *0.04306* | *1.12* |
| *13* | *rs79990454* | *32901430* | *C* | *0.1434* | *0.13* | *0.04189* | *1.121* |
| *13* | *rs79347017* | *32902067* | *G* | *0.1434* | *0.13* | *0.04189* | *1.121* |
| *13* | *rs2126042* | *32903685* | *T* | *0.04008* | *0.04858* | *0.04086* | *0.8177* |
| *13* | *rs11571638* | *32905628* | *A* | *0.1437* | *0.13* | *0.03803* | *1.123* |
| *13* | *rs11147486* | *32905998* | *G* | *0.1437* | *0.13* | *0.03803* | *1.123* |
| *13* | *rs766173* | *32906480* | *C* | *0.1437* | *0.13* | *0.03803* | *1.123* |
| *13* | *rs1801439* | *32906980* | *G* | *0.1437* | *0.13* | *0.03803* | *1.123* |
| *13* | *rs11571643* | *32907767* | *T* | *0.1437* | *0.13* | *0.03758* | *1.123* |
| *13* | *rs11571650* | *32910056* | *G* | *0.1437* | *0.13* | *0.03758* | *1.123* |
| *13* | *rs11571651* | *32910351* | *T* | *0.1437* | *0.13* | *0.03758* | *1.123* |
| *13* | *rs1801499* | *32910721* | *C* | *0.1437* | *0.13* | *0.03758* | *1.123* |
| *13* | *rs1799944* | *32911463* | *G* | *0.1437* | *0.1303* | *0.04181* | *1.12* |
| *13* | *rs4942439* | *32918941* | *G* | *0.04008* | *0.04898* | *0.03288* | *0.8107* |
| *13* | *rs4942440* | *32918967* | *A* | *0.04008* | *0.04898* | *0.03288* | *0.8107* |
| *13* | *rs4942443* | *32919526* | *T* | *0.04008* | *0.04898* | *0.03288* | *0.8107* |
| *13* | *rs4942448* | *32923690* | *T* | *0.03511* | *0.0451* | *0.01264* | *0.7703* |
| *13* | *rs11571695* | *32923988* | *G* | *0.1428* | *0.1297* | *0.0473* | *1.118* |
| *13* | *rs1853521* | *32925257* | *A* | *0.03498* | *0.04501* | *0.01256* | *0.7692* |
| *13* | *rs9943890* | *32928112* | *A* | *0.03433* | *0.04492* | *0.008014* | *0.7559* |
| *13* | *rs9943888* | *32928202* | *G* | *0.04178* | *0.05549* | *0.001877* | *0.7421* |
| *13* | *rs1460817* | *32928404* | *C* | *0.03433* | *0.04492* | *0.008014* | *0.7559* |
| *13* | *rs11147489* | *32929478* | *T* | *0.1435* | *0.1301* | *0.04149* | *1.121* |
| *13* | *rs9534259* | *32935406* | *C* | *0.03465* | *0.04484* | *0.01061* | *0.7645* |
| *13* | *rs11571722* | *32938173* | *C* | *0.1435* | *0.1301* | *0.04217* | *1.12* |
| *13* | *rs9534269* | *32939286* | *G* | *0.03465* | *0.04485* | *0.01054* | *0.7643* |
| *13* | *rs11571734* | *32940888* | *T* | *0.03465* | *0.04479* | *0.01101* | *0.7655* |
| *13* | *rs75649145* | *32943135* | *T* | *0.1436* | *0.1301* | *0.04* | *1.122* |
| *13* | *rs11571739* | *32943782* | *T* | *0.1436* | *0.1301* | *0.04046* | *1.121* |
| *13* | *rs11571742* | *32944098* | *A* | *0.03467* | *0.04475* | *0.0115* | *0.7667* |
| *13* | *rs10492395* | *32945537* | *T* | *0.03467* | *0.04475* | *0.0115* | *0.7667* |
| *13* | *rs573014* | *32945932* | *T* | *0.1789* | *0.2001* | *0.006784* | *0.8708* |
| *13* | *rs9526148* | *32951596* | *C* | *0.03467* | *0.04479* | *0.01122* | *0.766* |
| *13* | *rs10870659* | *32956109* | *A* | *0.03467* | *0.04475* | *0.01147* | *0.7666* |
| *13* | *rs9534344* | *32956283* | *A* | *0.03481* | *0.04477* | *0.01276* | *0.7696* |
| *13* | *rs7334543* | *32973276* | *G* | *0.03501* | *0.04467* | *0.01536* | *0.7758* |

Supplementary Table S3. Characteristics of study participants from three populations

| **Data** | |  | **Korean *BRCAX* (KOHBRA)** | **Asian high-risk (BCAC)** | **European high-risk (BCAC)** |
| --- | --- | --- | --- | --- | --- |
| **Controls** | | | | | |
|  | No. of participants |  | 5,979 | 3,612 | 10,214 |
|  | Age, mean±sd^a^ |  | 55.06 ± 10.19 | 42.56 ± 8.80 | 54.94 ± 11.66 |
| **Cases** | | | | | |
|  | No. of participants |  | 1,469 | 1,482 | 9,902 |
|  | Age, mean±sd^a^ |  | 40.24 ± 9.13 | 50.61 ± 22.29 | 49.51 ± 11.58 |
|  | Age of breast cancer onset | ≤40 | 1,037 (70.6%) | 603 (40.7%) | 3,107 (31.4%) |
|  |  | 41-45 | 143 (9.7%) | 591 (39.9%) | 2,673 (27.0%) |
|  |  | >45 | 289 (19.7%) | 288 (19.4%) | 4,122 (41.6%) |
|  | Family history of breast cancer in first degree relatives | Yes | 441 (30.0%) | 390 (26.3%) | 5,400 (54.5%) |
|  |  | No | 1,023 (69.6%) | 1,066 (71.9%) | 3,397 (34.3%) |
|  |  | Unknown | 5 (0.3%) | 26 (1.8%) | 1,104 (11.1%) |

^a^ sd: standard deviation

Supplementary Table S4. Meta-analysis of 15 genomic susceptibility loci by receptor status

| **SNP** | **Estrogen receptor** | | | | |  | **Progesterone receptor** | | | | |  | **HER2 receptor** | | | | |  | **Triple-negative (n=223/113)^a^** | |
| --- | --- | --- | --- | --- | --- | --- | --- | --- | --- | --- | --- | --- | --- | --- | --- | --- | --- | --- | --- | --- |
|  | **Positive (n=891/753)^a^** | | **Negative (n=465/382)^a^** | | **P_hetero_** |  | **Positive (n=879/703)^a^** | | **Negative (n=496/332)^a^** | | **P_hetero_** |  | **Positive (n=275/261)^a^** | | **Negative (n=924/424)^a^** | | **P_hetero_** |  |  |  |
|  | **P-value** | **OR** | **P-value** | **OR** |  |  | **P-value** | **OR** | **P-value** | **OR** |  |  | **P-value** | **OR** | **P-value** | **OR** |  |  | **P-value** | **OR** |
| rs60538652 | **1.87.E-07** | **1.28** | 0.320 | 1.07 | **0.021** |  | **1.91.E-07** | **1.29** | 0.302 | 1.07 | **0.018** |  | 0.107 | 1.14 | 0.120 | 1.12 | 0.875 |  | 0.750 | 0.97 |
| rs11154838 | 4.02.E-05 | 1.25 | 4.22.E-04 | 1.29 | 0.719 |  | 2.01.E-05 | 1.27 | 7.21.E-04 | 1.27 | 0.968 |  | 4.61.E-04 | 1.40 | 9.16.E-05 | 1.42 | 0.915 |  | 0.118 | 1.18 |
| rs9383936 | 6.07.E-07 | 1.24 | **8.79.E-14** | **1.53** | **0.004** |  | 7.12.E-07 | 1.25 | **7.82.E-12** | **1.48** | **0.022** |  | 8.42.E-08 | 1.48 | 1.38.E-05 | 1.32 | 0.245 |  | 1.05.E-05 | 1.46 |
| rs10953105 | 0.234 | 1.21 | 3.34.E-04 | 1.39 | 0.447 |  | 5.72.E-04 | 1.27 | 7.74.E-04 | 1.36 | 0.538 |  | 0.017 | 1.32 | 0.799 | 1.08 | 0.551 |  | 0.765 | 1.10 |
| rs2350923 | 0.202 | 1.20 | 0.580 | 1.08 | 0.576 |  | 0.270 | 1.15 | 0.273 | 1.17 | 0.935 |  | 0.452 | 1.17 | 0.345 | 1.18 | 0.969 |  | 0.036 | 1.22 |
| rs78545330 | 3.56.E-06 | 1.25 | 1.24.E-03 | 1.23 | 0.835 |  | 2.84.E-04 | 1.20 | 2.00.E-06 | 1.34 | 0.146 |  | 8.75.E-04 | 1.31 | 3.18.E-04 | 1.29 | 0.867 |  | 2.63.E-03 | 1.33 |
| rs10814070 | 0.264 | 1.11 | 0.415 | 1.17 | 0.796 |  | 0.480 | 1.09 | 0.422 | 1.16 | 0.763 |  | 0.954 | 1.00 | 0.227 | 1.08 | 0.398 |  | 0.240 | 1.37 |
| rs2912774 | **5.33.E-11** | **1.30** | 0.381 | 1.05 | **0.001** |  | **5.76.E-11** | **1.31** | 0.193 | 1.07 | **0.003** |  | 3.31.E-03 | 1.22 | 9.47.E-04 | 1.22 | 0.951 |  | 0.435 | 0.94 |
| rs9418690 | 1.24.E-05 | 1.27 | 0.026 | 1.18 | 0.383 |  | 0.322 | 1.16 | 2.87.E-03 | 1.24 | 0.693 |  | 0.012 | 1.27 | 0.047 | 1.19 | 0.605 |  | 0.157 | 1.16 |
| rs4964006 | 0.351 | 1.45 | 0.045 | 1.35 | 0.873 |  | 0.162 | 1.66 | 0.104 | 1.27 | 0.499 |  | 0.013 | 1.65 | 0.382 | 1.42 | 0.737 |  | 0.689 | 1.09 |
| rs67129489 | **5.01.E-08** | **1.44** | 0.413 | 1.08 | **0.015** |  | **2.84.E-07** | **1.42** | 0.335 | 1.09 | **0.024** |  | 5.31.E-03 | 1.36 | 0.026 | 1.27 | 0.668 |  | 0.204 | 0.82 |
| rs278050 | 7.46.E-05 | 1.22 | 1.14.E-04 | 1.29 | 0.526 |  | 1.10.E-04 | 1.22 | 1.20.E-05 | 1.34 | 0.303 |  | 0.027 | 1.21 | 0.027 | 1.19 | 0.877 |  | 2.97.E-03 | 1.34 |
| rs4784227 | 5.80.E-10 | 1.32 | 4.35.E-06 | 1.31 | 0.937 |  | 3.97.E-11 | 1.35 | 3.49.E-04 | 1.24 | 0.246 |  | 3.29.E-03 | 1.24 | 1.73.E-03 | 1.23 | 0.978 |  | 2.87.E-03 | 1.30 |
| rs4969001 | 0.878 | 1.03 | 0.555 | 1.15 | 0.730 |  | 0.843 | 1.05 | 0.596 | 1.12 | 0.598 |  | 0.978 | 0.99 | 0.565 | 1.12 | 0.618 |  | 0.731 | 1.17 |
| rs73107564 | 0.367 | 1.22 | 0.565 | 1.10 | 0.694 |  | 0.427 | 1.19 | 0.486 | 1.14 | 0.874 |  | 0.160 | 1.16 | 0.157 | 1.13 | 0.889 |  | 0.217 | 1.16 |

^a^ Numbers of Korean BRCAX (KOHBRA) and Asian high-risk cases (BCAC)

**Supplementary Table S5. Functional annotation of correlated SNPs (R^2^>0.8) with three novel SNPs using HaploReg**

| **SNP** | **Pos (hg19)** | **LD (R²)** | **Ref** | | **Alt** | **AFR** | **AMR** | **ASN** | **EUR** | **Promoter histone marks** | **Enhancer histone marks** | **DNase** | **Proteins bound** | **Motifs changed** | **Function** |
| --- | --- | --- | --- | --- | --- | --- | --- | --- | --- | --- | --- | --- | --- | --- | --- |
| ***PDE7B (Chr6)*** | |  |  | |  |  |  |  |  |  |  |  |  |  |  |
| rs6905776 | 136284300 | 0.92 | | G | A | 0.8 | 0.7 | 0.7 | 0.64 |  | LNG, STRM, MUS, BRN, BONE | BRST |  | CDP | intron |
| **rs11154838** | 136290942 | 1 | | C | T | 0.2 | 0.3 | 0.29 | 0.35 |  |  |  |  | BATF,Bach1 |  |
| rs9399176 | 136303107 | 0.8 | | C | T | 0.3 | 0.3 | 0.24 | 0.36 |  |  |  |  | DMRT2,DMRT3,HDAC2 |  |
| rs9402781 | 136330868 | 0.87 | | T | G | 0.3 | 0.3 | 0.31 | 0.36 |  |  |  |  |  |  |
| rs9389358 | 136331396 | 0.87 | | G | A | 0.3 | 0.3 | 0.31 | 0.36 |  |  |  |  | Cdx2,Evi-1 |  |
| rs12174235 | 136333103 | 0.86 | | T | C | 0.2 | 0.3 | 0.31 | 0.36 | LNG, STRM, BRN, BONE | ESDR, FAT, BLD, STRM, MUS, SKIN, OVRY, PLCNT, LNG, CRVX | LNG,MUS,LNG |  | FAC1,Foxd1,Foxf1,Foxf2,Foxj1,Foxj2,Foxk1,Foxl1,Foxo,Foxq1,HDAC2,Pou5f1,Sox |  |
| ***CDKN2B-AS1 (Chr9)*** | |  | |  |  |  |  |  |  |  |  |  |  |  |  |
| rs3731211 | 21986847 | 0.89 | | T | A | 0.7 | 0.8 | 0.75 | 0.7 | ESDR, LNG, FAT, STRM, BRN, SKIN, CRVX, VAS, BLD, BONE | FAT, MUS, BLD, BRN, LNG | ESDR,SKIN,CRVX,MUS,MUS,VAS,LNG | RFX5,TBP,MAFK | HNF1 | intron  intron |
| rs7036656 | 21990457 | 0.9 | | C | T | 0.72 | 0.8 | 0.75 | 0.7 | ESC, IPSC, FAT, STRM, SKIN, BRN, LNG | ESC, IPSC, BLD, BRST, MUS, VAS, BONE | ESDR,ESDR,ESDR,ESC,BLD,BLD,SKIN,SKIN,SKIN,SKIN,VAS,SKIN,LNG |  | DMRT5,DMRT7,RFX5 |  |
| **rs78545330** | 21995941 | 1 | | T | A | 0.26 | 0.1 | 0.24 | 0.08 | ESC, ESDR, LNG, IPSC, FAT, STRM, BRST, BLD, MUS, BRN, SKIN, LIV, GI, PANC, PLCNT, HRT, OVRY, SPLN, CRVX, VAS, BONE | ESDR, BLD, GI, THYM, PLCNT | ESC,ESDR,ESDR,ESDR,ESC,IPSC,BRST,BLD,BLD,BLD,BLD,BLD,SKIN,SKIN,SKIN,SKIN,MUS,MUS,PLCNT,GI,THYM,OVRY,PANC,MUS,GI,CRVX,LIV,BRST,MUS,VAS,BLD,BRN,SKIN,LNG | CTCF | Hsf,LUN-1 |  |
| rs113646886 | 21996645 | 1 | | CCT | C | 0.25 | 0.1 | 0.24 | 0.08 | ESC, IPSC, SKIN, BLD, CRVX, BRN, LNG | ESC, ESDR, STRM, BRST, BLD | ESC,ESDR,ESDR,ESDR,ESC,IPSC,IPSC,BLD,CRVX | ELK4,POL2 | ATF3,CACD,Irf,Klf4,Klf7,NF-kappaB,SP1,Sp4,TATA |  |
| rs2811712 | 21998035 | 0.98 | | G | A | 0.75 | 0.9 | 0.76 | 0.91 |  | ESC, IPSC, STRM, SKIN, CRVX, BRN |  |  | Dlx2,Dlx3,HNF1,Hltf,Ik-2,Nkx3,Nkx6-1,Pou3f2,Prrx2,Sox,TATA |  |
| rs3218010 | 21998733 | 0.86 | | T | C | 0 | 0 | 0.22 | 0 |  | ESC |  |  | Evi-1,Irf,ZNF263 |  |
| rs141283124 | 21998892 | 0.98 | | AAC | A | 0.25 | 0.1 | 0.24 | 0.08 |  | ESC |  |  | Barhl1,Barx1,Barx2,Bsx,DMRT2,Dbx2,Dlx2,Foxj1,Foxo,Foxp1,Gbx1,Hmx,Homez,Hoxd8,Nkx6-1,Pou6f1,Sox,Zfp105 |  |
| rs3218007 | 21999800 | 0.98 | | C | T | 0.25 | 0.1 | 0.24 | 0.08 |  | BLD, CRVX |  |  | Dbx2,Hoxd8,Pou2f2,Sox |  |
| rs3218005 | 22000247 | 0.98 | | T | C | 0.25 | 0.1 | 0.24 | 0.08 |  | BLD, CRVX | SKIN |  | CTCF,LXR |  |
| rs3218002 | 22000841 | 0.98 | | G | A | 0.25 | 0.1 | 0.24 | 0.08 | BLD | CRVX |  |  | Ets,GR,Nanog,Sox |  |
| rs3217995 | 22002556 | 0.84 | | TG | T | 0 | 0 | 0.22 | 0 |  | BLD, CRVX |  |  | AP-1,PRDM1 |  |
| rs181031884 | 22003887 | 0.86 | | A | T | 0 | 0 | 0.22 | 0 | BLD | STRM, BLD, CRVX |  |  | Foxp1,HDAC2,HMG-IY,Mef2,Pax-4,Zfp105 | 3'-UTR |
| rs974336 | 22006348 | 0.94 | | C | T | 0.27 | 0.1 | 0.25 | 0.08 | ESC, ESDR, LNG, IPSC, FAT, STRM, SKIN, BRN, GI, HRT, BONE | ESDR, BRST, BLD, GI, PANC, PLCNT, HRT, MUS, SPLN, SKIN | ESDR,ESDR,IPSC,IPSC,BRST,BLD,SKIN,BRN | CTCF,CEBPB,SUZ12 | Brachyury,Hoxa3 | intron |
| rs2285327 | 22007048 | 0.83 | | T | C | 0 | 0 | 0.22 | 0 | ESDR, FAT, STRM, MUS, SKIN, GI, CRVX, BRST, BRN, LNG | BRST, BLD |  | TFIIIC110 | Foxa,Foxj2,HNF1,HNF4 | intron |
| rs3808845 | 22010575 | 0.83 | | G | A | 0 | 0 | 0.22 | 0 |  | FAT, STRM, MUS, BRST, BONE | SKIN,MUS |  | Mef2,Zfp105 |  |
| rs3808846 | 22010946 | 0.83 | | G | A | 0 | 0 | 0.22 | 0 |  | FAT, STRM, BLD, GI, SKIN, BONE | BLD,SKIN,GI,THYM,OVRY,VAS | GATA1 | TFIIA |  |
| rs36084073 | 22012789 | 0.94 | | GA | G | 0.24 | 0.1 | 0.24 | 0.08 |  |  |  |  | ATF3,Cdx2,Hoxa10,Hoxa9,Hoxc10,Hoxc9,Hoxd10,Maf,p300 |  |
| rs116876910 | 22014411 | 0.83 | | G | A | 0 | 0 | 0.22 | 0 |  |  |  |  | Myf,Rad21 |  |
| rs149401414 | 22018125 | 0.83 | | C | G | 0 | 0 | 0.22 | 0 |  |  |  |  | ERalpha-a,GCNF,RXRA |  |
| rs138417214 | 22018437 | 0.82 | | A | G | 0 | 0 | 0.23 | 0.01 |  |  |  |  | ATF3,CTCF,HEN1,LXR,Rad21,SREBP |  |
| rs76219447 | 22018718 | 0.83 | | C | G | 0 | 0 | 0.22 | 0 |  |  |  |  | Elf3 |  |
| rs117418282 | 22022454 | 0.83 | | C | T | 0 | 0 | 0.22 | 0 |  |  |  | SETDB1 | PLZF |  |
| rs7039304 | 22022786 | 0.82 | | T | A | 0 | 0 | 0.22 | 0 |  |  |  | SETDB1 | Gbx1,Hoxa5 |  |
| rs76774391 | 22023775 | 0.83 | | G | C | 0 | 0 | 0.22 | 0 |  |  |  |  | LXR,Mef2 |  |
| rs76810097 | 22027714 | 0.81 | | C | T | 0 | 0 | 0.22 | 0 |  | BLD | IPSC |  | Mef2,Pax-2,Pax-3,Pax-5,ZEB1 |  |
| rs79666073 | 22031440 | 0.81 | | G | A | 0 | 0 | 0.22 | 0 |  | FAT, BLD, BRST, SKIN |  |  |  |  |
| rs77706751 | 22031441 | 0.81 | | C | T | 0 | 0 | 0.22 | 0 |  | FAT, BLD, BRST, SKIN |  |  | SIX5 |  |
| rs74599268 | 22031778 | 0.8 | | A | T | 0 | 0 | 0.22 | 0 |  | FAT, BRST, BLD, SKIN | BLD,SKIN |  | Sox |  |
| rs1333035 | 22044059 | 0.8 | | G | A | 0.75 | 0.9 | 0.78 | 0.91 |  | FAT, SKIN, BLD, BRST, VAS, BRN, BONE |  | MAFF,MAFK | AP-1 |  |
| **Near *UBL3/LINC00297 (Chr13)*** | | | |  |  |  |  |  |  |  |  |  |  |  |  |
| rs278046 | 30428389 | 0.94 | | C | T | 0.6 | 0.8 | 0.81 | 0.77 |  | SKIN |  |  | Myc,Rad21 | intergenic |
| rs278045 | 30428486 | 0.83 | | C | T | 0.9 | 0.8 | 0.79 | 0.9 |  |  |  |  |  |  |
| rs17631187 | 30433847 | 0.82 | | T | C | 0 | 0.1 | 0.17 | 0.07 |  | FAT, BLD, PLCNT, BONE |  |  | Arid3a,Gfi1,INSM1,Mef2 |  |
| rs149407523 | 30436214 | 0.82 | | GC | G | 0 | 0.1 | 0.17 | 0.07 | BONE | ESDR, LNG, FAT, STRM, MUS, SKIN, PLCNT, LIV, BRST, BRN | ESDR,ESDR,ESDR,LNG,BRST,SKIN,SKIN,SKIN,PLCNT,LNG,BRST,MUS,MUS,BRN,SKIN,SKIN,LNG |  | AP-1,TCF12 |  |
| rs278052 | 30436359 | 0.99 | | C | T | 0.6 | 0.8 | 0.81 | 0.77 | BONE | ESDR, LNG, FAT, STRM, MUS, SKIN, PLCNT, LIV, BRST, BRN | ESDR,LNG,BRST,SKIN,PLCNT,MUS,MUS,BRN,SKIN,LNG |  | Nkx2,Nkx3,Pou2f2 |  |
| rs278051 | 30436549 | 0.99 | | C | T | 1 | 0.9 | 0.81 | 0.91 | BONE | ESDR, LNG, FAT, STRM, MUS, SKIN, PLCNT, BRST, BRN |  |  | GR,Pbx3,RXRA,Zfp691 |  |
| **rs278050** | 30436968 | 1 | | C | A | 0.6 | 0.8 | 0.81 | 0.76 |  | LNG, FAT, PLCNT, BRST, MUS, SKIN, BONE |  |  |  |  |
| rs9550500 | 30438085 | 0.89 | | T | C | 0.1 | 0.2 | 0.18 | 0.09 |  |  |  |  | Obox3 |  |

Supplementary Table S6. eQTL findings of three novel SNPs and the nearest gene from GTEx data

| **Gene-SNP** | **P-value** | **Effect Size** | **T-Statistic** | **Standard Error** | **Tissue** |
| --- | --- | --- | --- | --- | --- |
| ***PDE7B***  ***-***  **rs11154838** | **0.001** | **0.27** | **3.3** | **0.082** | **Artery - Aorta** |
|  | 0.057 | -0.14 | -1.9 | 0.073 | Whole Blood |
|  | 0.12 | -0.13 | -1.6 | 0.082 | Brain - Anterior cingulate cortex |
|  | 0.12 | -0.22 | -1.6 | 0.14 | Prostate |
|  | 0.13 | 0.1 | 1.5 | 0.067 | Brain - Nucleus accumbens |
|  | 0.13 | 0.15 | 1.5 | 0.098 | Heart - Atrial Appendage |
|  | 0.13 | 0.13 | 1.5 | 0.085 | Heart - Left Ventricle |
|  | 0.14 | -0.085 | -1.5 | 0.057 | Stomach |
|  | 0.18 | 0.061 | 1.3 | 0.046 | Cells - Transformed fibroblasts |
|  | 0.19 | -0.1 | -1.3 | 0.076 | Esophagus - Mucosa |
|  | 0.19 | 0.077 | 1.3 | 0.059 | Nerve - Tibial |
|  | 0.19 | 0.17 | 1.3 | 0.13 | Ovary |
|  | 0.2 | 0.11 | 1.3 | 0.087 | Artery - Coronary |
|  | 0.2 | 0.096 | 1.3 | 0.074 | Breast - Mammary Tissue |
|  | 0.21 | -0.077 | -1.3 | 0.061 | Thyroid |
|  | 0.22 | 0.087 | 1.2 | 0.07 | Skin - Not Sun Exposed |
|  | 0.28 | 0.065 | 1.1 | 0.059 | Artery - Tibial |
|  | 0.29 | -0.074 | -1.1 | 0.07 | Testis |
|  | 0.32 | 0.11 | 1 | 0.11 | Pituitary |
|  | 0.36 | -0.073 | -0.91 | 0.08 | Esophagus - Muscularis |
|  | 0.37 | -0.067 | -0.91 | 0.073 | Brain - Hippocampus |
|  | 0.38 | -0.05 | -0.87 | 0.057 | Skin - Sun Exposed |
|  | 0.4 | 0.069 | 0.84 | 0.082 | Brain - Putamen |
|  | 0.41 | -0.04 | -0.83 | 0.048 | Muscle - Skeletal |
|  | 0.42 | -0.073 | -0.81 | 0.09 | Brain - Cortex |
|  | 0.43 | -0.09 | -0.79 | 0.11 | Brain - Cerebellar Hemisphere |
|  | 0.45 | 0.1 | 0.77 | 0.14 | Spleen |
|  | 0.46 | -0.083 | -0.75 | 0.11 | Liver |
|  | 0.57 | -0.07 | -0.57 | 0.12 | Brain - Frontal Cortex |
|  | 0.58 | -0.077 | -0.55 | 0.14 | Brain - Hypothalamus |
|  | 0.64 | 0.027 | 0.47 | 0.058 | Adipose - Visceral |
|  | 0.67 | -0.043 | -0.43 | 0.1 | Brain - Cerebellum |
|  | 0.71 | 0.032 | 0.38 | 0.085 | Vagina |
|  | 0.73 | 0.033 | 0.35 | 0.096 | Esophagus - Gastroesophageal Junction |
|  | 0.76 | 0.034 | 0.31 | 0.11 | Small Intestine - Terminal Ileum |
|  | 0.81 | 0.025 | 0.24 | 0.1 | Adrenal Gland |
|  | 0.81 | -0.039 | -0.25 | 0.16 | Uterus |
|  | 0.85 | 0.0096 | 0.19 | 0.049 | Brain - Caudate |
|  | 0.85 | 0.011 | 0.19 | 0.06 | Lung |
|  | 0.87 | -0.019 | -0.16 | 0.12 | Pancreas |
|  | 0.88 | -0.0085 | -0.16 | 0.055 | Adipose - Subcutaneous |
|  | 0.92 | -0.009 | -0.1 | 0.089 | Colon - Sigmoid |
| ***CDKN2B***  ***-***  **rs78545330** | **1.70E-13** | **-0.44** | **-7.7** | **0.057** | **Whole Blood** |
|  | **0.0098** | **-0.45** | **-2.6** | **0.17** | **Spleen** |
|  | **0.026** | **0.54** | **2.3** | **0.24** | **Brain - Spinal cord** |
|  | **0.027** | **-0.2** | **-2.3** | **0.087** | **Vagina** |
|  | **0.028** | **-0.15** | **-2.2** | **0.069** | **Small Intestine - Terminal Ileum** |
|  | **0.03** | **-0.37** | **-2.2** | **0.17** | **Brain - Hippocampus** |
|  | **0.045** | **-0.11** | **-2** | **0.054** | **Esophagus - Mucosa** |
|  | 0.051 | 0.41 | 2 | 0.21 | Brain - Hypothalamus |
|  | 0.057 | 0.26 | 1.9 | 0.14 | Brain - Cortex |
|  | 0.057 | -0.12 | -1.9 | 0.063 | Skin - Sun Exposed |
|  | 0.064 | -0.29 | -1.9 | 0.15 | Brain - Frontal Cortex |
|  | 0.13 | 0.085 | 1.5 | 0.056 | Adipose - Subcutaneous |
|  | 0.16 | -0.24 | -1.4 | 0.16 | Uterus |
|  | 0.19 | -0.13 | -1.3 | 0.096 | Esophagus - Gastroesophageal Junction |
|  | 0.22 | -0.13 | -1.2 | 0.1 | Minor Salivary Gland |
|  | 0.23 | 0.27 | 1.2 | 0.22 | Brain - Amygdala |
|  | 0.25 | -0.11 | -1.2 | 0.094 | Stomach |
|  | 0.3 | -0.11 | -1 | 0.11 | Heart - Atrial Appendage |
|  | 0.3 | 0.11 | 1.1 | 0.11 | Ovary |
|  | 0.34 | 0.051 | 0.96 | 0.053 | Breast - Mammary Tissue |
|  | 0.35 | 0.07 | 0.93 | 0.075 | Artery - Tibial |
|  | 0.35 | 0.11 | 0.93 | 0.12 | Brain - Cerebellum |
|  | 0.35 | -0.064 | -0.93 | 0.068 | Esophagus - Muscularis |
|  | 0.36 | -0.063 | -0.92 | 0.069 | Skin - Not Sun Exposed |
|  | 0.4 | 0.089 | 0.85 | 0.1 | Pancreas |
|  | 0.41 | -0.098 | -0.83 | 0.12 | Colon - Sigmoid |
|  | 0.43 | 0.073 | 0.79 | 0.092 | Heart - Left Ventricle |
|  | 0.46 | 0.048 | 0.74 | 0.064 | Muscle - Skeletal |
|  | 0.48 | -0.08 | -0.71 | 0.11 | Adrenal Gland |
|  | 0.5 | -0.056 | -0.68 | 0.083 | Artery - Aorta |
|  | 0.54 | -0.038 | -0.62 | 0.061 | Cells - Transformed fibroblasts |
|  | 0.56 | -0.035 | -0.59 | 0.06 | Testis |
|  | 0.58 | -0.077 | -0.55 | 0.14 | Brain - Nucleus accumbens |
|  | 0.59 | -0.083 | -0.54 | 0.15 | Brain - Caudate |
|  | 0.64 | 0.033 | 0.47 | 0.071 | Adipose - Visceral |
|  | 0.64 | -0.11 | -0.47 | 0.23 | Brain - Substantia nigra |
|  | 0.65 | -0.1 | -0.46 | 0.22 | Cells - EBV-transformed lymphocytes |
|  | 0.68 | 0.049 | 0.42 | 0.12 | Pituitary |
|  | 0.78 | -0.038 | -0.27 | 0.14 | Liver |
|  | 0.86 | 0.023 | 0.18 | 0.13 | Prostate |
|  | 0.88 | -0.012 | -0.15 | 0.08 | Lung |
|  | 0.9 | -0.021 | -0.12 | 0.17 | Brain - Putamen |
|  | 0.91 | -0.016 | -0.12 | 0.14 | Brain - Anterior cingulate cortex |
|  | 0.95 | -0.0085 | -0.06 | 0.14 | Brain - Cerebellar Hemisphere |
|  | 0.96 | -0.0068 | -0.048 | 0.14 | Artery - Coronary |
|  | 0.96 | -0.0029 | -0.048 | 0.06 | Nerve - Tibial |
|  | 0.99 | 0.0015 | 0.018 | 0.085 | Thyroid |
| ***UBL3***  ***-***  **rs278050** | **0.011** | **0.13** | **2.6** | **0.049** | **Testis** |
|  | **0.018** | **-0.1** | **-2.4** | **0.043** | **Stomach** |
|  | **0.04** | **0.089** | **2.1** | **0.043** | **Cells - Transformed fibroblasts** |
|  | 0.056 | 0.22 | 1.9 | 0.11 | Ovary |
|  | 0.06 | -0.21 | -1.9 | 0.11 | Vagina |
|  | 0.092 | -0.064 | -1.7 | 0.038 | Lung |
|  | 0.14 | -0.12 | -1.5 | 0.083 | Artery - Coronary |
|  | 0.19 | -0.034 | -1.3 | 0.025 | Whole Blood |
|  | 0.22 | 0.066 | 1.2 | 0.054 | Heart - Left Ventricle |
|  | 0.25 | -0.069 | -1.2 | 0.06 | Brain - Cerebellum |
|  | 0.25 | -0.059 | -1.2 | 0.051 | Skin - Not Sun Exposed |
|  | 0.26 | 0.077 | 1.1 | 0.068 | Brain - Hippocampus |
|  | 0.31 | -0.12 | -1 | 0.12 | Brain - Hypothalamus |
|  | 0.31 | -0.056 | -1 | 0.055 | Heart - Atrial Appendage |
|  | 0.32 | 0.089 | 0.99 | 0.09 | Brain - Frontal Cortex |
|  | 0.47 | 0.025 | 0.72 | 0.034 | Esophagus - Mucosa |
|  | 0.5 | 0.046 | 0.67 | 0.069 | Esophagus - Gastroesophageal Junction |
|  | 0.54 | -0.02 | -0.62 | 0.033 | Skin - Sun Exposed |
|  | 0.54 | -0.029 | -0.62 | 0.047 | Thyroid |
|  | 0.55 | 0.046 | 0.6 | 0.076 | Brain - Caudate |
|  | 0.55 | 0.043 | 0.61 | 0.072 | Brain - Putamen |
|  | 0.57 | -0.022 | -0.57 | 0.038 | Adipose - Subcutaneous |
|  | 0.57 | 0.05 | 0.58 | 0.086 | Adrenal Gland |
|  | 0.57 | -0.023 | -0.57 | 0.039 | Artery - Tibial |
|  | 0.57 | 0.05 | 0.57 | 0.088 | Brain - Cerebellar Hemisphere |
|  | 0.59 | 0.027 | 0.55 | 0.05 | Breast - Mammary Tissue |
|  | 0.6 | -0.044 | -0.52 | 0.084 | Spleen |
|  | 0.62 | -0.018 | -0.49 | 0.037 | Muscle - Skeletal |
|  | 0.64 | 0.025 | 0.47 | 0.054 | Adipose - Visceral |
|  | 0.65 | 0.033 | 0.45 | 0.074 | Small Intestine - Terminal Ileum |
|  | 0.69 | -0.023 | -0.4 | 0.059 | Artery - Aorta |
|  | 0.71 | 0.013 | 0.37 | 0.036 | Nerve - Tibial |
|  | 0.72 | -0.016 | -0.36 | 0.044 | Esophagus - Muscularis |
|  | 0.74 | 0.025 | 0.33 | 0.075 | Brain - Nucleus accumbens |
|  | 0.76 | -0.044 | -0.31 | 0.14 | Uterus |
|  | 0.82 | 0.023 | 0.23 | 0.097 | Cells - EBV-transformed lymphocytes |
|  | 0.84 | -0.021 | -0.2 | 0.11 | Liver |
|  | 0.87 | -0.011 | -0.17 | 0.065 | Brain - Cortex |
|  | 0.88 | -0.017 | -0.15 | 0.11 | Prostate |
|  | 0.9 | -0.015 | -0.12 | 0.12 | Pituitary |
|  | 0.93 | 0.0073 | 0.089 | 0.082 | Brain - Anterior cingulate cortex |
|  | 0.93 | -0.008 | -0.091 | 0.087 | Colon - Sigmoid |
|  | 0.98 | 0.0011 | 0.024 | 0.046 | Pancreas |

Supplementary Table S7. Replication analysis of previously reported loci

| **Population** | **Position (hg19)** | **Nearby gene(s)** | **SNP** | **Risk allele** | **RAF^a^** | |  | **Meta-analysis of Published Studies** | |  | | **Korean *BRCAX* cases (KOHBRA)^b^** | | |  | | | **Asian high-risk cases (BCAC)^b^** | | | | |  | **European**  **high-risk cases (BCAC)^b^** | | |  |
| --- | --- | --- | --- | --- | --- | --- | --- | --- | --- | --- | --- | --- | --- | --- | --- | --- | --- | --- | --- | --- | --- | --- | --- | --- | --- | --- | --- |
|  |  |  |  |  | **EAS** | **EUR** |  | **P-value** | **OR** |  | | **P-value** | **OR** | **FRR** |  | | | **P-value** | **OR** | **FRR** | | |  | **P-value** | **OR** | **FRR** |  |
| **Asian, European  and other population  (Latino, Ashkenazi Jewish or Sardinian)** | 6:151948366 | *CCDC170, ESR1* | rs2046210 | A | 0.36 | 0.32 |  | 3.54.E-09 | 1.19 |  | | 1.14E-09 | 1.30 | 2.84% |  | | | 2.87.E-06 | 1.26 | 2.25% | | |  | 7.76.E-07 | 1.13 | 0.53% |  |
|  | 10:123346116 | *FGFR2* | rs2981575 | G | 0.43 | 0.44 |  | 1.00.E-08 | 1.28 |  | | 2.07E-08 | 1.27 | 2.34% |  | | | 1.13.E-02 | 1.13 | 0.62% | | |  | 1.98.E-37 | 1.35 | 3.72% |  |
|  | 16:52599188 | *CASC16, TOX3* | rs4784227 | T | 0.26 | 0.25 |  | 1.13.E-44 | 1.25 |  | | 1.87E-09 | 1.31 | 2.65% |  | | | 6.24.E-05 | 1.25 | 1.72% | | |  | 2.02.E-22 | 1.29 | 2.40% |  |
| **Asian, European** | 11:129473690 | *RPS27P20 - LINC01395, TMEM45B, BARX2* | rs7107217 | C | 0.35 | 0.53 |  | 5.00.E-07 | 1.08 |  | | 1.13E-02 | 1.12 | 0.50% |  | | | 5.28.E-02 | 1.10 |  | | |  | 5.09.E-03 | 1.07 | 0.18% |  |
|  | 12:14413931 | *GNAI2P1 - RPL30P11, ATF7IP* | rs12422552 | C | 0.27 | 0.29 |  | 4.00.E-08 | 1.05 |  | | 6.02E-05 | 1.22 | 1.44% |  | | | 1.09.E-01 | 1.09 |  | | |  | 1.14.E-01 | 1.04 |  |  |
| **Asian** | 1:87779217 | *LOC101927844 - LMO4* | rs12118297 | G | 0.73 | 0.80 |  | 4.00.E-08 | 1.10 |  | | 1.10E-01 | 1.07 |  |  | | | 1.65.E-02 | 1.14 | 0.56% | | |  | 7.72.E-03 | 1.09 | 0.18% |  |
|  | 1:203766331 | *ZC3H11A* | rs4951011 | G | 0.31 | 0.15 |  | 9.00.E-09 | 1.09 |  | | 2.22E-03 | 1.15 | 0.78% |  | | | 9.00.E-01 | 1.01 |  | | |  | 5.35.E-01 | 1.02 |  |  |
|  | 2:213296863 | *ERBB4* | rs13393577 | T | 0.96 | 0.90 |  | 9.00.E-14 | 1.53 |  | | 9.70E-01 | 1.00 |  |  | | | 2.63.E-01 | 1.17 |  | | |  | 2.20.E-01 | 1.06 |  |  |
|  | 3:150467808 | *SIAH2* | rs6788895 | G | 0.60 | 0.97 |  | 9.00.E-08 | 1.22 |  | | 2.06E-06 | 1.23 | 1.60% |  | | | 8.47.E-01 | 1.01 |  | | |  | 6.99.E-01 | 1.03 |  |  |
|  | 5:90732225 | *ARRDC3-AS1 - RAB5CP2* | rs10474352 | C | 0.51 | 0.86 |  | 2.00.E-09 | 1.09 |  | | 1.48E-01 | 1.06 |  |  | | | 4.19.E-01 | 0.95 |  | | |  | 4.09.E-03 | 1.12 | 0.24% |  |
|  | 6:152295613 | *CCDC170, ESR1* | rs9383951 | G | 0.93 | 1.00 |  | 2.00.E-06 | 1.14 |  | | 5.34E-01 | 1.05 |  |  | | | 7.66.E-01 | 0.97 |  | | |  | 3.54.E-01 | 1.25 |  |  |
|  | 6:149608874 | *TAB2* | rs9485372 | G | 0.58 | 0.84 |  | 4.00.E-12 | 1.11 |  | | 1.14E-02 | 1.11 | 0.45% |  | | | 9.44.E-03 | 1.14 | 0.67% | | |  | 3.15.E-02 | 1.07 | 0.09% |  |
|  | 7:130653851 | *LINC-PINT* | rs2048672 | C | 0.45 | 0.68 |  | 6.00.E-06 | 1.11 |  | | 2.38E-01 | 1.05 |  |  | | | 7.09.E-01 | 0.98 |  | | |  | 4.27.E-01 | 1.02 |  |  |
|  | 10:64251977 | *ZNF365* | rs10822013 | T | 0.51 | 0.50 |  | 6.00.E-09 | 1.12 |  | | 6.72E-04 | 1.15 | 0.84% |  | | | 2.97.E-01 | 1.05 |  | | |  | 4.71.E-03 | 1.07 | 0.17% |  |
|  | 15:91512067 | *PRC1, PRC1-AS1* | rs2290203 | G | 0.49 | 0.79 |  | 4.00.E-08 | 1.08 |  | | 8.66E-02 | 1.07 |  |  | | | 1.01.E-02 | 1.13 | 0.64% | | |  | 8.46.E-01 | 1.01 |  |  |
|  | 18:20776651 | *CABLES1* | rs11082321 | A | 0.16 | 0.07 |  | 7.00.E-07 | 1.08 |  | | 4.27E-02 | 1.10 | 0.23% |  | | | 9.23.E-01 | 1.01 |  | | |  | 4.73.E-01 | 0.94 |  |  |
|  | 21:36111201 | *LINC00160, LOC107985515* | rs16992204 | C | 0.14 | 0.00 |  | 5.00.E-08 | 1.13 |  | | 3.07E-03 | 1.17 | 0.59% |  | | | 4.99.E-01 | 1.06 |  | | |  | 5.24.E-01 | 1.27 |  |  |
|  | 22:39358037 | *APOBEC3A* | rs12628403 | C | 0.34 | 0.07 |  | 4.00.E-06 | 1.17 |  | | 1.80E-04 | 1.48 | 6.43% |  | | | 5.19.E-01 | 0.94 |  | | |  | 6.70.E-01 | 0.97 |  |  |
| **European, African** | 5:1279790 | *TERT* | rs10069690 | T | 0.17 | 0.28 |  | 3.82.E-06 | 1.10 |  | | 2.16E-04 | 1.24 | 1.22% |  | | | 1.28.E-01 | 1.10 |  | | |  | 3.45.E-05 | 1.11 | 0.41% |  |
|  | 6:82193109 | *LOC105377871* | rs17530068 | C | 0.25 | 0.23 |  | 7.60.E-04 | 1.07 |  | | 7.22E-02 | 1.09 |  |  | | | 8.53.E-01 | 1.01 |  | | |  | 1.61.E-03 | 1.09 | 0.23% |  |
|  | 10:80841148 | *ZMIZ1* | rs704010 | T | 0.31 | 0.41 |  | 1.66.E-18 | 1.08 |  | | 7.67E-02 | 1.09 |  |  | | | 1.84.E-01 | 1.07 |  | | |  | 9.45.E-05 | 1.10 | 0.34% |  |
|  | 10:81055626 | *ZMIZ1* | rs12355688 | T | 0.25 | 0.06 |  | 6.00.E-06 | 1.24 |  | | 3.65E-01 | 0.95 |  |  | | | 3.24.E-02 | 1.13 | 0.47% | | |  | 4.31.E-01 | 1.04 |  |  |
|  | 14:68699594 | *RAD51L1, RAD51B* | rs1314913 | T | 0.03 | 0.17 |  | 3.00.E-13 | 1.57 |  | | 4.46E-01 | 1.09 |  |  | | | 2.62.E-01 | 1.15 |  | | |  | 2.24.E-03 | 1.10 | 0.24% |  |
|  | 14:68973546 | *RAD51L1, RAD51B* | rs757369 | A | 0.55 | 0.47 |  | 2.00.E-06 | 1.67 |  | | 1.24E-01 | 1.08 |  |  | | | 9.54.E-01 | 1.00 |  | | |  | 7.03.E-02 | 0.96 |  |  |
|  | 19:17392894 | *BABAM1, ANKLE1, C19orf62, ABHD8* | rs8100241 | G | 0.69 | 0.43 |  | 4.00.E-08 | 1.14 |  | | 2.09E-03 | 1.15 | 0.64% |  | | | 4.84.E-01 | 1.04 |  | | |  | 7.01.E-02 | 1.04 |  |  |
| **European**  **European**  **European** | 1:10566215 | *PEX14* | rs616488 | A | 0.68 | 0.67 |  | 2.20.E-14 | 1.07 |  | | 3.65E-01 | 1.04 |  |  | | | 2.47.E-01 | 1.06 |  | | |  | 2.41.E-03 | 1.08 | 0.20% |  |
|  | 1:84622513 | *LOC107985046, PRKACB* | rs903263 | T | 0.38 | 0.54 |  | 1.00.E-06 | 1.27 |  | | 8.73E-01 | 1.01 |  |  | | | 6.47.E-01 | 0.98 |  | | |  | 9.03.E-01 | 1.00 |  |  |
|  | 1:114448389 | *BCL2L15, AP4B1, DCLRE1B, HIPK1, PTPN22* | rs11552449 | T | 0.59 | 0.19 |  | 2.00.E-08 | 1.07 |  | | 6.45E-01 | 0.98 |  |  | | | NA | NA |  | | |  | NA | NA |  |  |
|  | 1:121280613 | *EMBP1* | rs11249433 | G | 0.03 | 0.43 |  | 7.14.E-24 | 1.10 |  | | 1.34E-01 | 1.17 |  |  | | | 8.76.E-01 | 1.02 |  | | |  | 3.78.E-05 | 1.10 | 0.38% |  |
|  | 1:202187176 | *LGR6, UBE2T, PTPN7* | rs6678914 | G | 0.73 | 0.57 |  | 1.00.E-08 | 1.10 |  | | 6.33E-01 | 1.03 |  |  | | | 1.87.E-01 | 0.93 |  | | |  | 8.23.E-01 | 0.99 |  |  |
|  | 1:204516025 | *MDM4* | rs2290854 | A | 0.30 | 0.31 |  | 1.00.E-07 | 1.13 |  | | 9.41E-02 | 0.93 |  |  | | | 8.92.E-01 | 0.99 |  | | |  | 1.82.E-01 | 1.03 |  |  |
|  | 2:19320803 | *LOC105373455 - MIR4757* | rs12710696 | T | 0.31 | 0.34 |  | 5.00.E-08 | 1.10 |  | | 1.40E-02 | 1.11 | 0.44% |  | | | 6.52.E-01 | 1.02 |  | | |  | 1.98.E-02 | 1.06 | 0.12% |  |
|  | 2:121245122 | *LINC01101 - LOC105373585* | rs4849887 | C | 0.79 | 0.90 |  | 4.00.E-11 | 1.10 |  | | 8.46E-01 | 0.99 |  |  | | | 4.33.E-01 | 1.05 |  | | |  | 7.06.E-04 | 1.14 | 0.24% |  |
|  | 2:172972971 | *METAP1D, DLX1, DLX2* | rs2016394 | G | 0.81 | 0.54 |  | 1.00.E-08 | 1.05 |  | | 2.11E-01 | 0.93 |  |  | | | 3.11.E-02 | 0.88 |  | | |  | 1.60.E-04 | 1.09 | 0.31% |  |
|  | 2:174212894 | *LOC100289479 - CDCA7* | rs1550623 | A | 1.00 | 0.85 |  | 3.00.E-08 | 1.06 |  | | 4.98E-01 | 0.58 |  |  | | | 1.52.E-01 | 1.31 |  | | |  | 2.29.E-05 | 1.14 | 0.35% |  |
|  | 2:217905832 | *LOC105373874, LOC101928278* | rs13387042 | A | 0.10 | 0.52 |  | 4.99.E-14 | 1.16 |  | | 1.99E-01 | 1.09 |  |  | | | 2.13.E-01 | 1.09 |  | | |  | 2.31.E-11 | 1.16 | 0.95% |  |
|  | 2:218296508 | *DIRC3* | rs16857609 | T | 0.58 | 0.27 |  | 1.00.E-15 | 1.08 |  | | 1.65E-02 | 1.11 | 0.42% |  | | | 1.23.E-01 | 1.08 |  | | |  | 1.25.E-01 | 1.04 |  |  |
|  | 3:4742276 | *ITPR1, EGOT* | rs6762644 | G | 0.07 | 0.35 |  | 2.00.E-12 | 1.07 |  | | 3.08E-01 | 1.08 |  |  | | | 2.08.E-01 | 0.90 |  | | |  | 2.36.E-03 | 1.07 | 0.20% |  |
|  | 3:27416013 | *NEK10, SLC4A7* | rs4973768 | T | 0.18 | 0.48 |  | 8.36.E-09 | 1.11 |  | | 7.81E-01 | 1.01 |  |  | | | 6.80.E-02 | 1.11 |  | | |  | 3.01.E-11 | 1.16 | 0.97% |  |
|  | 3:30682939 | *TGFBR2* | rs12493607 | C | 0.71 | 0.34 |  | 2.00.E-08 | 1.06 |  | | 4.66E-01 | 1.03 |  |  | | | 6.65.E-01 | 1.02 |  | | |  | 8.00.E-02 | 1.04 |  |  |
|  | 4:106084778 | *TET2* | rs9790517 | T | 0.63 | 0.21 |  | 4.00.E-08 | 1.05 |  | | 6.53E-02 | 0.92 |  |  | | | 3.72.E-01 | 0.96 |  | | |  | 1.64.E-01 | 1.04 |  |  |
|  | 4:175846426 | *ADAM29* | rs6828523 | C | 0.76 | 0.89 |  | 4.00.E-16 | 1.11 |  | | 1.32E-01 | 1.08 |  |  | | | 1.63.E-01 | 1.08 |  | | |  | 1.88.E-03 | 1.12 | 0.19% |  |
|  | 5:10467702 | *ROPN1L* | rs1092913 | A | 0.69 | 0.10 |  | 2.00.E-06 | 1.45 |  | | 3.90E-01 | 0.96 |  |  | | | 8.11.E-01 | 1.02 |  | | |  | 7.58.E-01 | 0.98 |  |  |
|  | 5:44662515 | *LOC102723839 - RN7SL383P* | rs4415084 | T | 0.55 | 0.41 |  | 8.00.E-11 | 1.17 |  | | 1.85E-02 | 1.11 | 0.42% |  | | | 4.41.E-01 | 1.04 |  | | |  | 1.83.E-08 | 1.14 | 0.72% |  |
|  | 5:45285718 | *HCN1* | rs981782 | C | 0.35 | 0.47 |  | 9.00.E-06 | 1.04 |  | | 5.43E-01 | 0.97 |  |  | | | 6.50.E-03 | 1.15 | 0.80% | | |  | 8.87.E-03 | 0.94 |  |  |
|  | 5:56168712 | *MAP3K1* | rs2229882 | T | 0.06 | 0.05 |  | 1.00.E-14 | 1.45 |  | | 4.42E-04 | 1.30 | 0.86% |  | | | 3.35.E-02 | 1.22 | 0.49% | | |  | 9.17.E-07 | 1.28 | 0.58% |  |
|  | 5:58184061 | *RAB3C - PDE4D* | rs10472076 | C | 0.30 | 0.36 |  | 3.00.E-08 | 1.05 |  | | 8.30E-01 | 1.01 |  |  | | | 8.44.E-02 | 0.91 |  | | |  | 1.43.E-01 | 1.04 |  |  |
|  | 5:58337481 | *PDE4D* | rs1353747 | T | 1.00 | 0.92 |  | 3.00.E-08 | 1.09 |  | | NA | NA |  |  | | | 3.41.E-01 | 1.36 |  | | |  | 1.25.E-01 | 1.06 |  |  |
|  | 5:158244083 | *EBF1* | rs1432679 | C | 0.61 | 0.45 |  | 6.82.E-16 | 1.07 |  | | 3.42E-02 | 1.10 | 0.35% |  | | | 8.58.E-02 | 1.09 |  | | |  | 1.10.E-06 | 1.12 | 0.53% |  |
|  | 6:1318878 | *FOXQ1 - LINC01394* | rs11242675 | T | 0.42 | 0.66 |  | 7.00.E-09 | 1.06 |  | | 9.88E-01 | 1.00 |  |  | | | 1.80.E-01 | 0.94 |  | | |  | 4.56.E-03 | 1.07 | 0.17% |  |
|  | 6:13722523 | *RANBP9 - MCUR1* | rs204247 | G | 0.62 | 0.42 |  | 8.00.E-09 | 1.05 |  | | 7.34E-01 | 1.02 |  |  | | | 3.02.E-01 | 1.05 |  | | |  | 4.52.E-02 | 1.05 | 0.09% |  |
|  | 7:8552614 | *NXPH1* | rs765855 | A | 0.28 | 0.34 |  | 4.00.E-06 | NA |  | | 1.00E-01 | 1.08 |  |  | | | 9.12.E-01 | 1.01 |  | | |  | 4.41.E-01 | 0.98 |  |  |
|  | 7:144074929 | *ARHGEF5, NOBOX* | rs720475 | G | 0.96 | 0.71 |  | 7.00.E-11 | 1.06 |  | | 8.80E-01 | 1.02 |  |  | | | 4.09.E-02 | 1.27 | 0.30% | | |  | 2.02.E-05 | 1.12 | 0.41% |  |
|  | 8:29509616 | *RPL17P33 - LINC00589* | rs9693444 | A | 0.30 | 0.35 |  | 9.00.E-14 | 1.07 |  | | 5.33E-02 | 1.09 |  |  | | | 7.66.E-01 | 1.02 |  | | |  | 5.01.E-02 | 1.05 |  |  |
|  | 8:76230301 | *HIGD1AP6 - PKMP4* | rs6472903 | T | 0.97 | 0.84 |  | 2.00.E-17 | 1.10 |  | | 1.68E-02 | 0.78 |  |  | | | 4.12.E-01 | 1.11 |  | | |  | 2.16.E-05 | 1.14 | 0.36% |  |
|  | 8:76417937 | *HNF4G* | rs2943559 | G | 0.08 | 0.07 |  | 6.00.E-15 | 1.13 |  | | 3.55E-01 | 1.07 |  |  | | | 8.10.E-02 | 0.86 |  | | |  | 4.49.E-04 | 1.16 | 0.26% |  |
|  | 8:128387852 | *CASC21, CASC8* | rs1562430 | T | 0.82 | 0.59 |  | 1.32.E-16 | 1.16 |  | | 6.10E-03 | 1.19 | 0.69% |  | | | 7.80.E-01 | 0.98 |  | | |  | 3.25.E-07 | 1.13 | 0.56% |  |
|  | 8:129194641 | *MYC, MIR1208, RN7SKP226* | rs11780156 | T | 0.19 | 0.19 |  | 3.00.E-11 | 1.07 |  | | 8.65E-01 | 1.01 |  |  | | | 5.87.E-01 | 0.97 |  | | |  | 8.93.E-03 | 1.08 | 0.17% |  |
|  | 9:22062134 | *CDKN2B-AS1* | rs1011970 | T | 0.11 | 0.16 |  | 1.36.E-07 | 1.07 |  | | 7.48E-01 | 0.97 |  |  | | | 7.35.E-02 | 1.16 |  | | |  | 2.86.E-02 | 1.07 | 0.10% |  |
|  | 9:110306115 | *LOC105376206 - LOC105376205* | rs10759243 | A | 0.45 | 0.31 |  | 1.00.E-08 | 1.06 |  | | 3.59E-01 | 1.04 |  |  | | | 2.30.E-02 | 1.12 | 0.50% | | |  | 1.21.E-02 | 1.07 | 0.15% |  |
|  | 9:110888478 | *LOC105376214* | rs865686 | T | 0.92 | 0.63 |  | 1.57.E-49 | 1.12 |  | | 2.57E-01 | 1.10 |  |  | | | 9.36.E-01 | 0.99 |  | | |  | 1.34.E-08 | 1.14 | 0.68% |  |
|  | 10:5886734 | *GDI2 - TRV-TAC3-1* | rs2380205 | C | 0.87 | 0.56 |  | 5.00.E-07 | 1.06 |  | | 7.76E-01 | 0.98 |  |  | | | 9.09.E-01 | 1.01 |  | | |  | 6.37.E-01 | 1.01 |  |  |
|  | 10:22032942 | *LOC107984214* | rs7072776 | A | 0.05 | 0.29 |  | 4.00.E-14 | 1.07 |  | | 9.18E-01 | 1.01 |  |  | | | 9.26.E-01 | 0.99 |  | | |  | 3.51.E-03 | 1.08 | 0.19% |  |
|  | 10:22315843 | *DNAJC1 - ADIPOR1P1* | rs11814448 | C | 0.01 | 0.02 |  | 9.00.E-16 | 1.26 |  | | 6.53E-01 | 0.75 |  |  | | | 2.35.E-01 | 1.26 |  | | |  | 8.57.E-02 | 1.15 |  |  |
|  | 10:64278682 | *ZNF365* | rs10995190 | G | 0.98 | 0.85 |  | 1.13.E-11 | 1.15 |  | | 3.60E-01 | 1.15 |  |  | | | 2.56.E-02 | 0.72 |  | | |  | 3.63.E-04 | 1.12 | 0.26% |  |
|  | 10:114773927 | *TCF7L2* | rs7904519 | G | 0.03 | 0.50 |  | 3.00.E-08 | 1.06 |  | | 6.18E-01 | 0.93 |  |  | | | 9.33.E-01 | 1.01 |  | | |  | 1.80.E-04 | 1.09 | 0.31% |  |
|  | 10:123093901 | *LOC105378523 - RN7SKP167* | rs11199914 | C | 0.62 | 0.68 |  | 2.00.E-08 | 1.05 |  | | 5.45E-02 | 1.09 |  |  | | | 7.10.E-01 | 1.02 |  | | |  | 2.39.E-02 | 1.06 | 0.11% |  |
|  | 10:123625190 | *FGFR2, ATE1* | rs10510102 | C | 0.18 | 0.18 |  | 2.00.E-06 | 1.12 |  | | 4.78E-01 | 1.04 |  |  | | | 4.37.E-01 | 0.95 |  | | |  | 9.04.E-01 | 1.00 |  |  |
|  | 11:1941946 | *LSP1, TNNT3* | rs909116 | T | 0.36 | 0.54 |  | 7.00.E-07 | 1.17 |  | | 7.83E-02 | 1.09 |  |  | | | 5.14.E-01 | 1.03 |  | | |  | 2.45.E-02 | 1.05 | 0.11% |  |
|  | 11:65583066 | *DKFZp761E198, OVOL1, SNX32, CFL1, MUS81* | rs3903072 | G | 0.77 | 0.54 |  | 9.00.E-12 | 1.05 |  | | 7.58E-01 | 1.02 |  |  | | | 5.78.E-02 | 1.12 |  | | |  | 9.17.E-06 | 1.11 | 0.42% |  |
|  | 11:69307695 | *CCND1, LINC01488* | rs537626 | C | 0.02 | 0.15 |  | 2.00.E-15 | 1.29 |  | | 9.94E-01 | 1.00 |  |  | | | 1.03.E-01 | 1.27 |  | | |  | 7.92.E-10 | 1.21 | 0.87% |  |
|  | 12:28155080 | *PTHLH - LOC105369710* | rs10771399 | A | 0.82 | 0.89 |  | 1.95.E-10 | 1.17 |  | | 4.67E-03 | 1.17 | 0.57% |  | | | 4.27.E-03 | 1.20 | 0.75% | | |  | 3.00.E-07 | 1.21 | 0.49% |  |
|  | 12:96027759 | *NTN4, USP44 - PGAM1P5* | rs17356907 | A | 0.74 | 0.71 |  | 3.77.E-27 | 1.10 |  | | 9.96E-01 | 1.00 |  |  | | | 2.93.E-02 | 1.13 | 0.45% | | |  | 6.15.E-04 | 1.09 | 0.25% |  |
|  | 12:115836522 | *LOC105370003* | rs1292011 | A | 0.77 | 0.59 |  | 9.00.E-22 | 1.09 |  | | 3.79E-01 | 1.04 |  |  | | | 4.02.E-02 | 1.12 | 0.37% | | |  | 2.11.E-06 | 1.12 | 0.49% |  |
|  | 14:37132769 | *PAX9, SLC25A21* | rs2236007 | G | 0.68 | 0.78 |  | 2.00.E-13 | 1.08 |  | | 8.40E-01 | 1.01 |  |  | | | 8.65.E-01 | 1.01 |  | | |  | 4.84.E-04 | 1.10 | 0.26% |  |
|  | 14:91841069 | *CCDC88C* | rs941764 | G | 0.14 | 0.35 |  | 4.00.E-10 | 1.06 |  | | 7.84E-01 | 1.02 |  |  | | | 5.59.E-01 | 1.04 |  | | |  | 5.03.E-04 | 1.09 | 0.28% |  |
|  | 16:53813367 | *FTO, MIR1972-2, KIAA1752* | rs17817449 | T | 0.83 | 0.59 |  | 2.65.E-12 | 1.08 |  | | 2.78E-02 | 1.16 | 0.48% |  | | | 3.37.E-01 | 1.06 |  | | |  | 2.31.E-02 | 1.05 | 0.11% |  |
|  | 16:80650805 | *CDYL2* | rs13329835 | G | 0.04 | 0.24 |  | 2.00.E-16 | 1.08 |  | | 8.46E-01 | 1.02 |  |  | | | 1.93.E-01 | 1.15 |  | | |  | 2.90.E-06 | 1.13 | 0.52% |  |
|  | 17:53056471 | *STXBP4, COX11* | rs6504950 | G | 0.90 | 0.73 |  | 2.00.E-13 | 1.06 |  | | 1.97E-02 | 1.26 | 0.70% |  | | | 8.51.E-01 | 1.01 |  | | |  | 2.10.E-02 | 1.06 | 0.11% |  |
|  | 18:24337424 | *PCAT18 - LOC105372035* | rs527616 | G | 0.74 | 0.60 |  | 2.00.E-10 | 1.05 |  | | 3.02E-01 | 0.93 |  |  | | | 4.40.E-01 | 1.04 |  | | |  | 8.34.E-02 | 1.04 |  |  |
|  | 18:24570667 | *CHST9* | rs1436904 | T | 0.51 | 0.59 |  | 3.00.E-08 | 1.04 |  | | 4.46E-01 | 1.03 |  |  | | | 2.76.E-01 | 1.05 |  | | |  | 1.45.E-03 | 1.08 | 0.22% |  |
|  | 19:18571141 | *SSBP4, ISYNA1, ELL* | rs4808801 | A | 0.78 | 0.66 |  | 5.00.E-15 | 1.08 |  | | 1.11E-01 | 1.08 |  |  | | | 6.88.E-01 | 0.98 |  | | |  | 4.43.E-09 | 1.15 | 0.73% |  |
|  | 19:44286513 | *C19orf61, KCNN4, LYPD5, ZNF283, LOC107987268* | rs3760982 | A | 0.19 | 0.46 |  | 2.00.E-10 | 1.06 |  | | 4.82E-01 | 1.04 |  |  | | | 1.52.E-02 | 0.86 |  | | |  | 1.73.E-01 | 1.03 |  |  |
|  | 19:52372976 | *ZNF577* | rs10411161 | T | 0.30 | 0.13 |  | 7.00.E-06 | 1.42 |  | | 9.09E-01 | 1.01 |  |  | | | 5.10.E-01 | 0.97 |  | | |  | 4.65.E-01 | 0.97 |  |  |
|  | 20:32588095 | *RALY, EIF2S2, ASIP* | rs2284378 | T | 0.17 | 0.29 |  | 1.00.E-08 | 1.16 |  | | 3.25E-01 | 0.95 |  |  | | | 9.63.E-01 | 1.00 |  | | |  | 3.51.E-01 | 1.02 |  |  |
|  | 21:16520832 | *NRIP1, LOC107985483, LOC105372739* | rs2823093 | G | 0.96 | 0.74 |  | 7.00.E-16 | 1.09 |  | | 7.74E-01 | 1.04 |  |  | | | 9.27.E-01 | 1.01 |  | | |  | 1.76.E-03 | 1.08 | 0.20% |  |
|  | 22:29621477 | *EMID1, RHBDD3, EWSR1, CHEK2* | rs132390 | C | 0.00 | 0.02 |  | 3.00.E-09 | 1.12 |  | | NA | NA |  |  | | | 9.34.E-01 | 0.96 |  | | |  | 4.09.E-02 | 1.13 | 0.07% |  |
|  | 22:40876234 | *MKL1* | rs6001930 | C | 0.24 | 0.10 |  | 3.64.E-21 | 1.12 |  | | 1.67E-01 | 1.06 |  |  | | | 5.08.E-01 | 1.04 |  | | |  | 1.04.E-06 | 1.19 | 0.54% |  |
| **African** | 14:88295600 | *LOC105370611 - GALC* | rs4322600 | G | 0.95 | 0.73 |  | 4.00.E-06 | 1.18 |  | | 2.54E-01 | 1.11 |  |  | | | 9.62.E-01 | 1.00 |  | | |  | 5.92.E-01 | 1.01 |  |  |
| **Ashkenazi Jewish** | 6:19443935 | *RNA5SP205 - LOC105374960* | rs16882214 | C | 0.86 | 0.85 |  | 2.00.E-06 | 1.43 |  | | 1.81E-01 | 1.12 |  |  | | | 1.99.E-01 | 0.90 |  | | |  | 3.40.E-01 | 0.97 |  |  |
|  | 6:127600630 | *ECHDC1, RNF146* | rs2180341 | G | 0.20 | 0.28 |  | 3.00.E-08 | 1.41 |  | | 6.39E-01 | 0.98 |  |  | | | 1.67.E-02 | 0.87 |  | | |  | 5.67.E-01 | 0.99 |  |  |
|  | 15:78269472 | *ADAMTS7P3* | rs12906542 | G | 0.13 | 0.21 |  | 7.00.E-07 | 2.00 |  | | 1.23E-03 | 1.37 | 2.33% |  | | | 7.51.E-01 | 0.97 |  | | |  | 9.28.E-01 | 1.00 |  |  |
| **Sum of %FRR (all SNPs with p-value <0.05)** | | | | | | | | | | | **29.79%** | | | | |  | **10.60%** | | | |  | **23.95%** | | | | | |

**^a^** RAF: risk allele frequency

^b^ FRR (familial recurrence risk) was estimated for replicated loci only (with p-value <0.05 and OR in the same direction with that from the previous study)

Supplementary Table S8. Replication analysis of the variant in breast cancer predisposition genes

| **Data** | **Gene** | **SNP** | **Chr** | **Position (hg19)** | **Function (or distance to gene for intergenic marker)** | **Minor Allele** | **MAF in cases^a^** | **MAF**  **in controls^a^** | **OR (95% CI)^b^** | ***P*-value** | **%FRR^c^** |
| --- | --- | --- | --- | --- | --- | --- | --- | --- | --- | --- | --- |
| **Korean BRCAX cases** | *BMPR1A* | rs80265064 | 10 | 88676265 | intronic | T | 0.066 | 0.081 | 0.8 (0.68-0.94) | 7.47.E-03 | 0.515% |
|  | *PTEN* | rs56014839 | 10 | 89663981 | intronic | A | 0.008 | 0.017 | 0.45 (0.29-0.71) | 4.59.E-04 | 0.84% |
|  | *CDH1* | rs17690554 | 16 | 68869510 | downstream (66 bp) | G | 0.153 | 0.174 | 0.86 (0.77-0.96) | 7.47.E-03 | 0.52% |
|  | *NF1* | rs3785956 | 17 | 29647707 | intronic | A | 0.357 | 0.324 | 1.16 (1.07-1.26) | 5.98.E-04 | 0.85% |
|  | *RAD51C* | rs302872 | 17 | 56773237 | intronic | G | 0.492 | 0.468 | 1.11 (1.03-1.21) | 9.96.E-03 | 0.50% |
|  | *BRIP1* | rs9630735 | 17 | 59943704 | downstream (2.8 Kb) | G | 0.460 | 0.445 | 1.11 (1.03-1.21) | 9.73.E-03 | 0.48% |
|  | *CHEK2* | rs3788408 | 22 | 29120797 | intronic | G | 0.113 | 0.097 | 1.19 (1.05-1.35) | 8.70.E-03 | 0.51% |
|  | **Sum of %FRR** | |  |  |  |  |  |  |  |  | **4.21%** |
| **Asian high-risk cases** | *MSH2* | rs148111763 | 2 | 47719393 | downstream (9.0 Kb) | G | 0.002 | 0.007 | 0.31 (0.13-0.75) | 9.89.E-03 | 0.60% |
|  | *BARD1* | rs2734674 | 2 | 215658895 | intronic | A | 0.207 | 0.236 | 0.86 (0.77-0.96) | 8.86.E-03 | 0.66% |
|  | *PMS2* | rs80044764 | 7 | 6018592 | intronic | A | 0.072 | 0.053 | 1.36 (1.08-1.7) | 8.18.E-03 | 1.05% |
|  |  | rs147261211 | 7 | 6046477 | intronic | A | 0.009 | 0.004 | 2.33 (1.29-4.2) | 5.21.E-03 | 1.19% |
|  | *FANCC* | rs78340532 | 9 | 97871838 | intronic | A | 0.027 | 0.032 | 0.63 (0.47-0.84) | 1.86.E-03 | 0.74% |
|  | *CDH1* | rs74025312 | 16 | 68856951 | intronic | G | 0.178 | 0.192 | 0.83 (0.74-0.94) | 3.90.E-03 | 0.77% |
|  | *TP53* | rs1800370 | 17 | 7579579 | **exonic** | T | 0.002 | 0.000 | 11.2 (2.17-57.95) | 3.96.E-03 | 4.82% |
|  | *RAD51D* | - | 17 | 33424465 | intronic | C | 0.005 | 0.003 | 2.91 (1.42-5.98) | 3.68.E-03 | 1.62% |
|  | *BRIP1* | rs72842985 | 17 | 59841070 | intronic | T | 0.163 | 0.177 | 0.84 (0.74-0.96) | 8.19.E-03 | 0.66% |
|  |  | rs12941248 | 17 | 59880581 | intronic | A | 0.364 | 0.408 | 0.85 (0.77-0.93) | 7.60.E-04 | 1.10% |
|  | **Sum of %FRR** | | |  |  |  |  |  |  |  | **13.21%** |
| **European high-risk cases** | *MSH6* | rs112432539 | 2 | 48015521 | intronic | A | 0.025 | 0.020 | 1.21 (1.05-1.39) | 7.86.E-03 | 0.14% |
|  | *BARD1* | rs280622 | 2 | 215590410 | upstream (2.9 Kb) | G | 0.007 | 0.005 | 1.53 (1.17-2) | 1.91.E-03 | 0.23% |
|  | *MLH1* | rs148314286 | 3 | 37039352 | intronic | C | 0.008 | 0.010 | 0.74 (0.59-0.92) | 8.05.E-03 | 0.12% |
|  | *PMS2* | rs11978631 | 7 | 6024038 | intronic | T | 0.061 | 0.072 | 0.83 (0.76-0.9) | 8.26.E-06 | 0.34% |
|  |  | rs12112229 | 7 | 6036515 | intronic | A | 0.259 | 0.242 | 1.09 (1.04-1.15) | 2.20.E-04 | 0.25% |
|  |  | rs149880598 | 7 | 6056139 | downstream (7.4 Kb) | T | 0.004 | 0.002 | 2.36 (1.53-3.64) | 1.03.E-04 | 0.48% |
|  | *XRCC2* | rs3218517 | 7 | 152350022 | intronic | A | 0.016 | 0.013 | 1.32 (1.11-1.58) | 1.92.E-03 | 0.23% |
|  | *NBN* | rs145654908 | 8 | 90980037 | intronic | T | 0.008 | 0.009 | 0.71 (0.56-0.92) | 9.03.E-03 | 0.12% |
|  | *FANCC* | rs117170968 | 9 | 97853064 | upstream (8.3 Kb) | T | 0.002 | 0.001 | 2.39 (1.39-4.1) | 1.57.E-03 | 0.32% |
|  |  | rs62560524 | 9 | 98017665 | intronic | T | 0.242 | 0.256 | 0.92 (0.88-0.96) | 6.03.E-04 | 0.22% |
|  | *BMPR1A* | rs61858633 | 10 | 88608947 | intronic | C | 0.016 | 0.020 | 0.81 (0.69-0.95) | 7.98.E-03 | 0.12% |
|  |  | rs116980028 | 10 | 88650626 | intronic | A | 0.001 | 0.002 | 0.38 (0.21-0.67) | 9.91.E-04 | 0.15% |
|  | *PTEN* | rs113598180 | 10 | 89614751 | upstream (8.4 Kb) | G | 0.002 | 0.002 | 2.02 (1.2-3.41) | 8.22.E-03 | 0.35% |
|  |  | rs146942279 | 10 | 89693992 | intronic | C | 0.000 | 0.000 | 0.04 (0-0.38) | 4.68.E-03 | 0.04% |
|  | *PALB2* | rs75617851 | 16 | 23604920 | upstream (9.6 Kb) | G | 0.015 | 0.016 | 0.78 (0.65-0.94) | 7.96.E-03 | 0.13% |
|  | *CDH1* | rs150045763 | 16 | 68778858 | intronic | C | 0.006 | 0.007 | 0.58 (0.44-0.76) | 8.91.E-05 | 0.21% |
|  | *NF1* | rs71372253 | 17 | 29413019 | upstream (8.9 Kb) | C | 0.047 | 0.047 | 0.84 (0.75-0.93) | 1.29.E-03 | 0.20% |
|  |  | rs12949394 | 17 | 29591152 | intronic | G | 0.051 | 0.052 | 0.83 (0.75-0.92) | 2.60.E-04 | 0.25% |
|  |  | rs144872859 | 17 | 29642511 | intronic | A | 0.001 | 0.001 | 4.53 (1.68-12.17) | 2.76.E-03 | 1.88% |
|  | *RAD51C* | - | 17 | 56769063 | upstream (0.9 Kb) | G | 0.001 | 0.001 | 0.35 (0.17-0.73) | 5.18.E-03 | 0.10% |
|  | *SMAD4* | rs145011178 | 18 | 48604513 | intronic | C | 0.001 | 0.002 | 0.45 (0.26-0.78) | 4.78.E-03 | 0.11% |
|  | *CHEK2* | rs62235680 | 22 | 29074976 | upstream (8.8 Kb) | C | 0.010 | 0.010 | 0.72 (0.57-0.89) | 3.16.E-03 | 0.14% |
|  |  | rs62235681 | 22 | 29080731 | upstream (3.0 Kb) | G | 0.009 | 0.005 | 2.02 (1.51-2.71) | 2.68.E-06 | 0.88% |
|  |  | rs17882647 | 22 | 29132640 | intronic | A | 0.014 | 0.015 | 0.78 (0.65-0.94) | 8.11.E-03 | 0.12% |
|  |  | rs17878974 | 22 | 29138715 | downstream (0.9 Kb) | A | 0.001 | 0.001 | 3.23 (1.33-7.87) | 9.80.E-03 | 0.75% |
|  | **Sum of %FRR** | | |  |  |  |  |  |  |  | **7.91%** |

^a^ MAF: Minor allele frequency

^b^ OR: Odds ratio, CI: Confidence interval

^c^ FRR: Familial recurrence risk


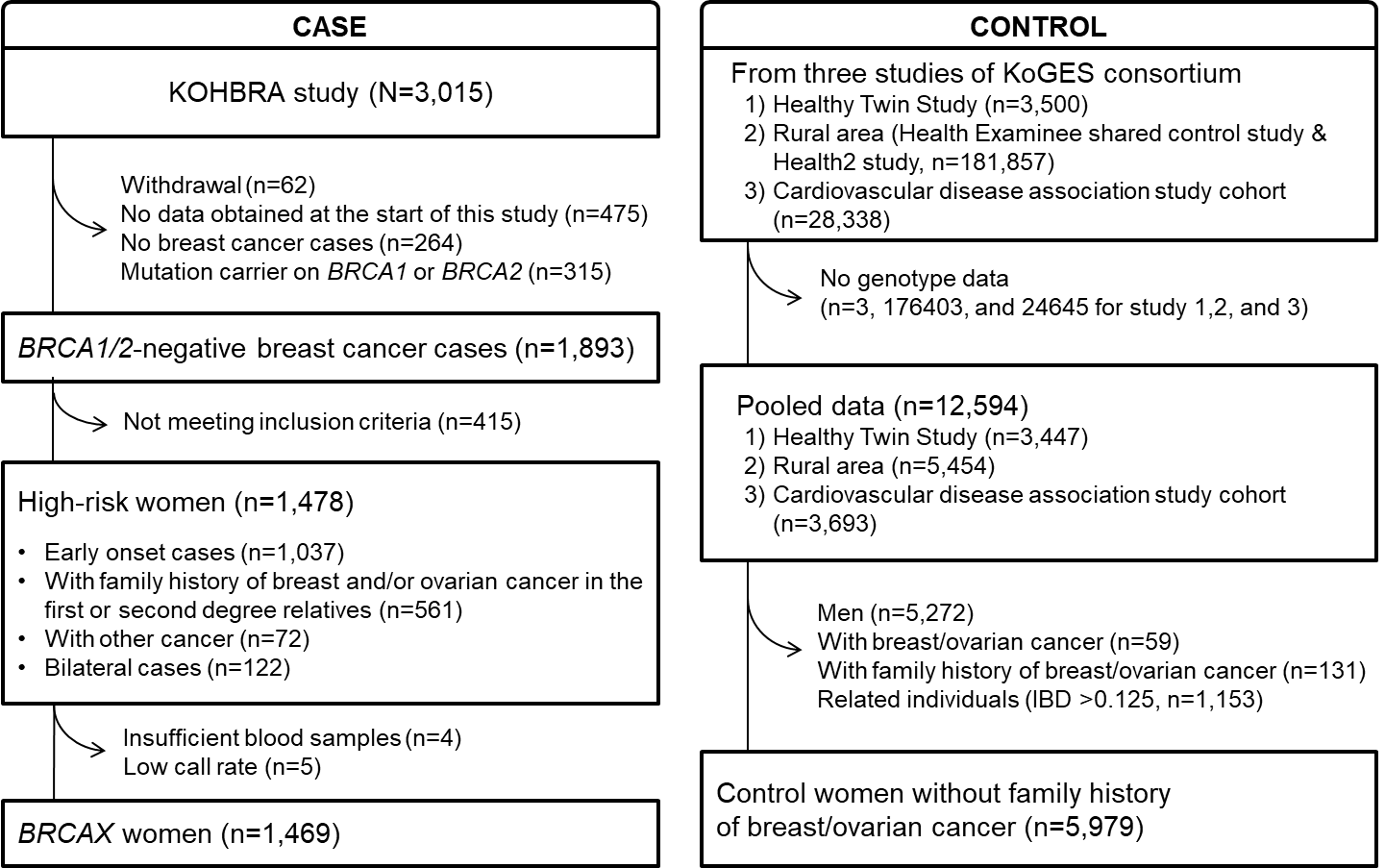


Supplementary Figure S1. Discovery set flowchart


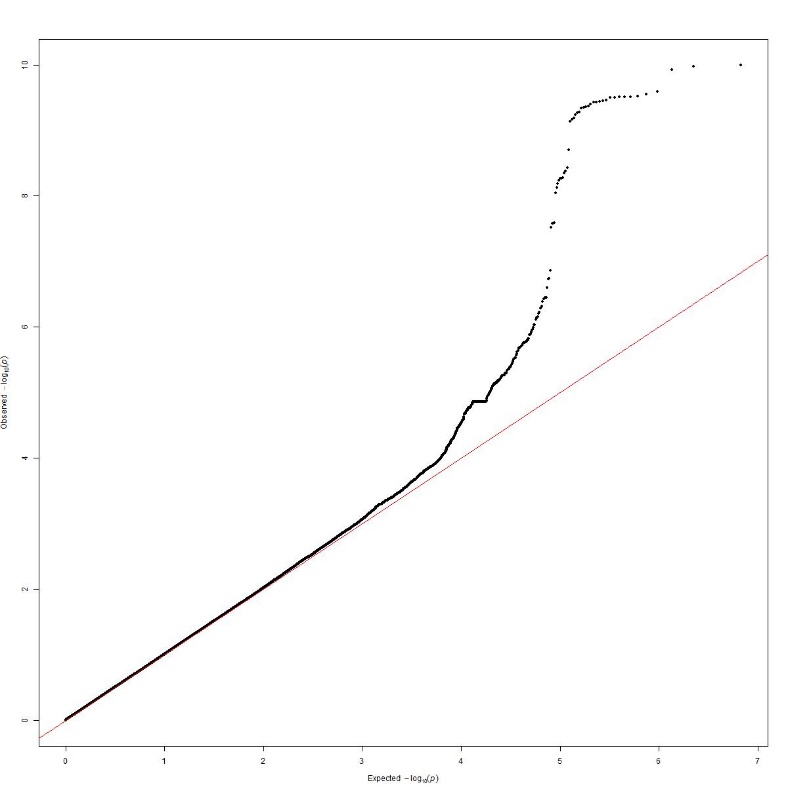
 Genomic inflation factor was 1.09 and *p*-values were corrected for the inflation factor.

Supplementary Figure S2. Quantile-quantile plot

**(a)**


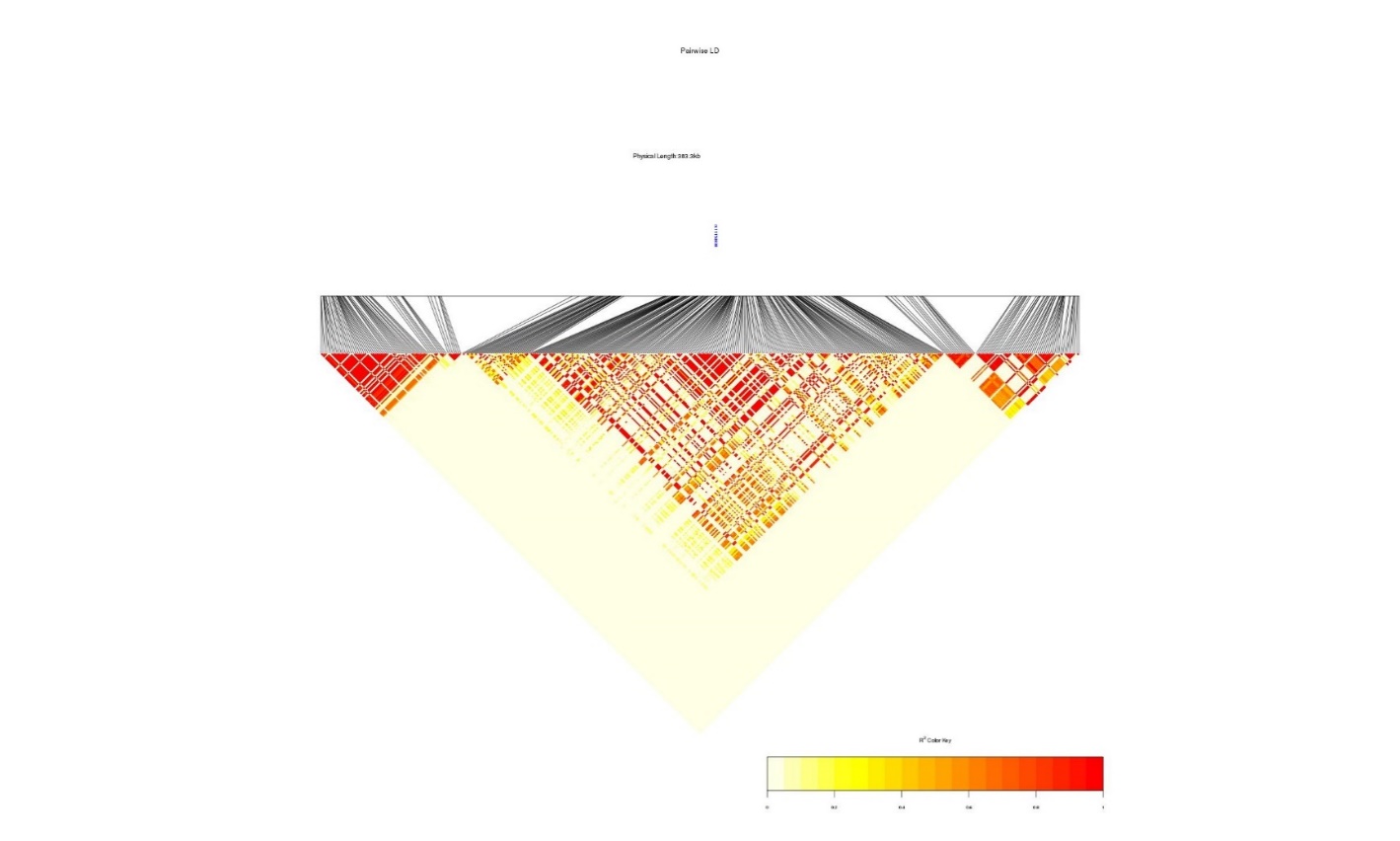

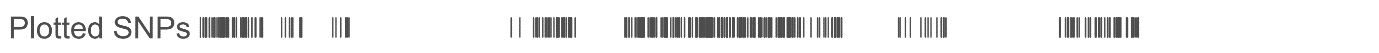

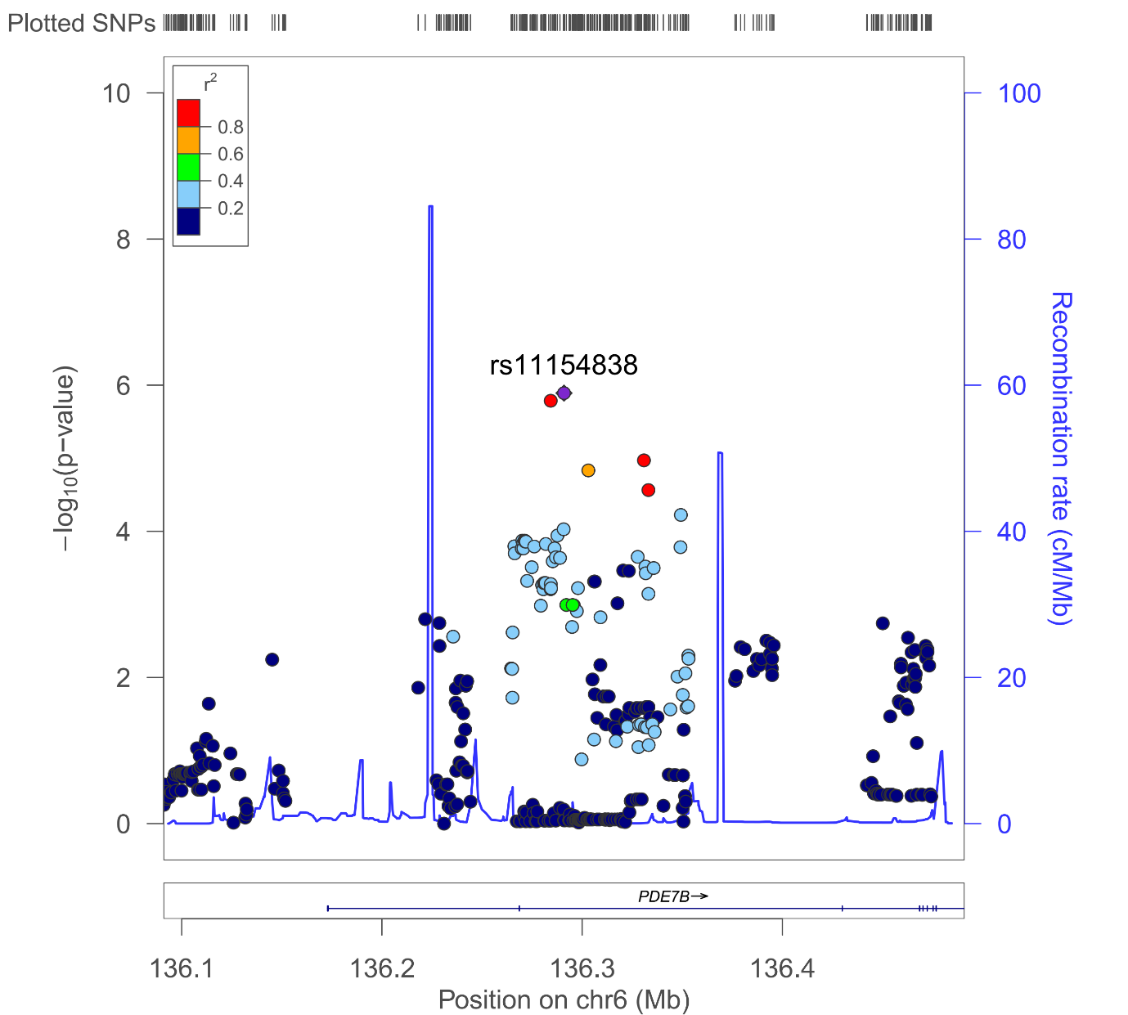

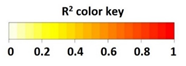


**(b)**


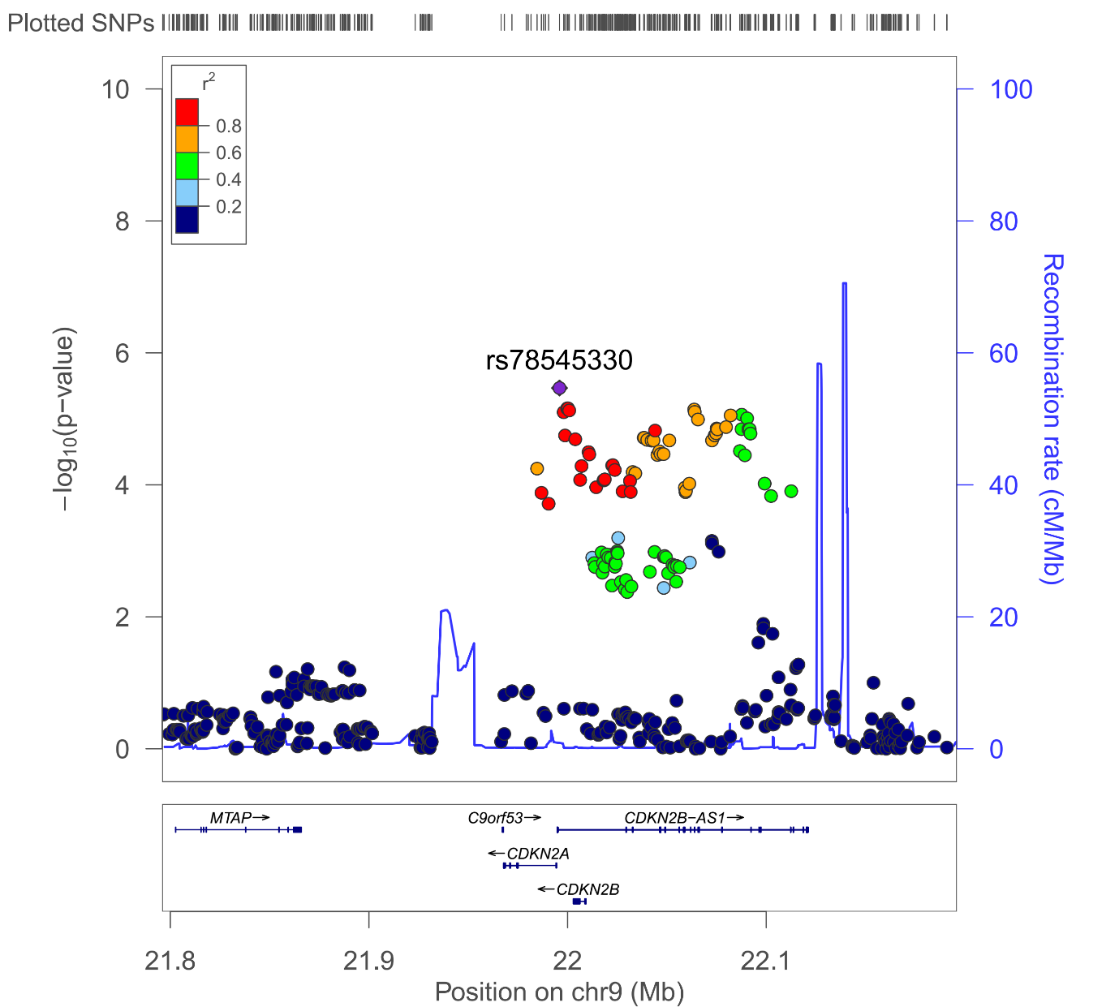

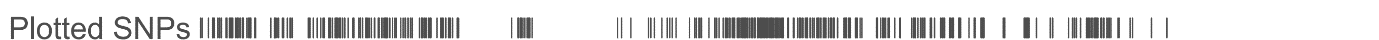

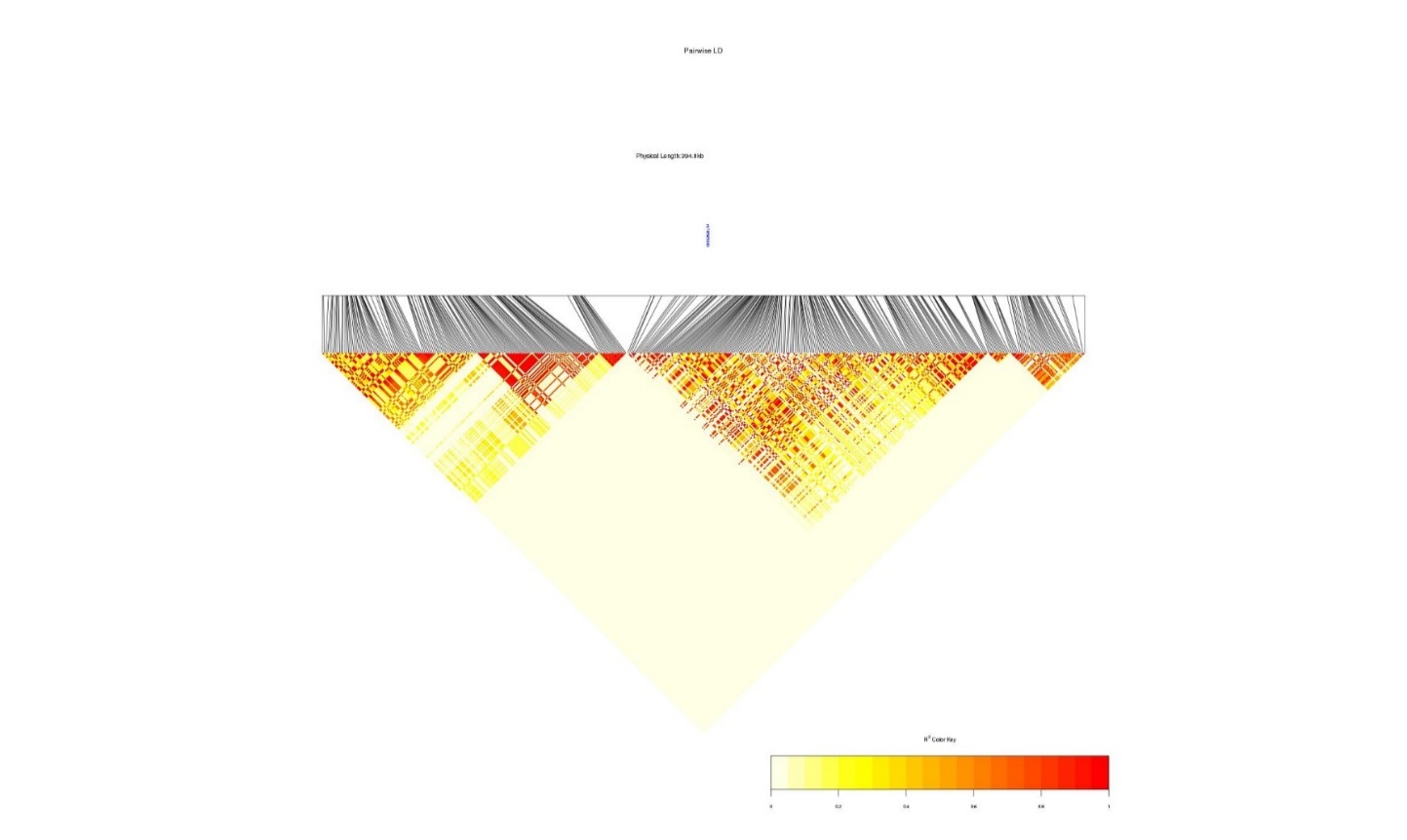

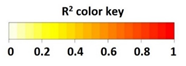


**(c)**


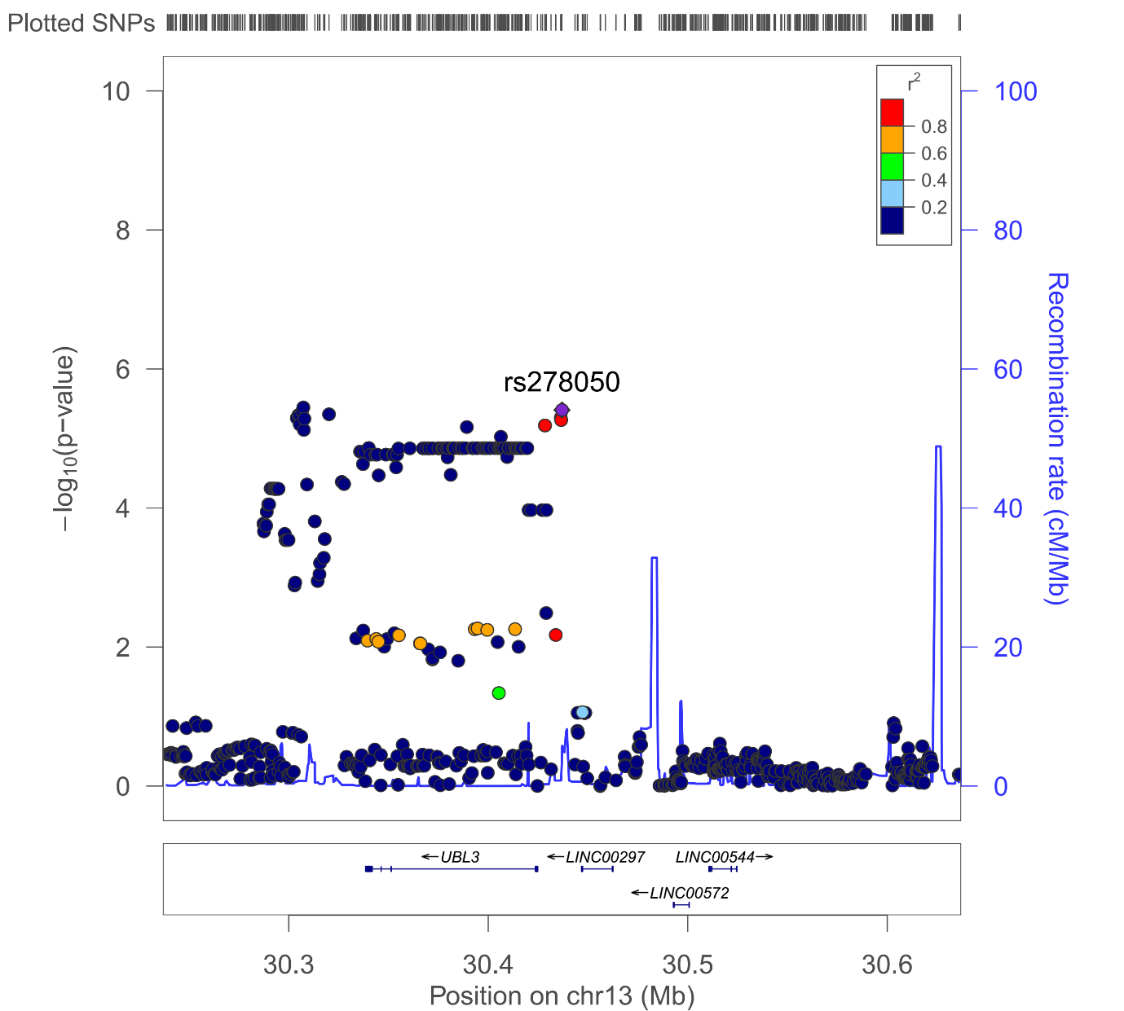

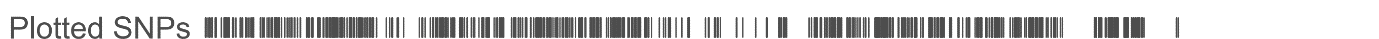

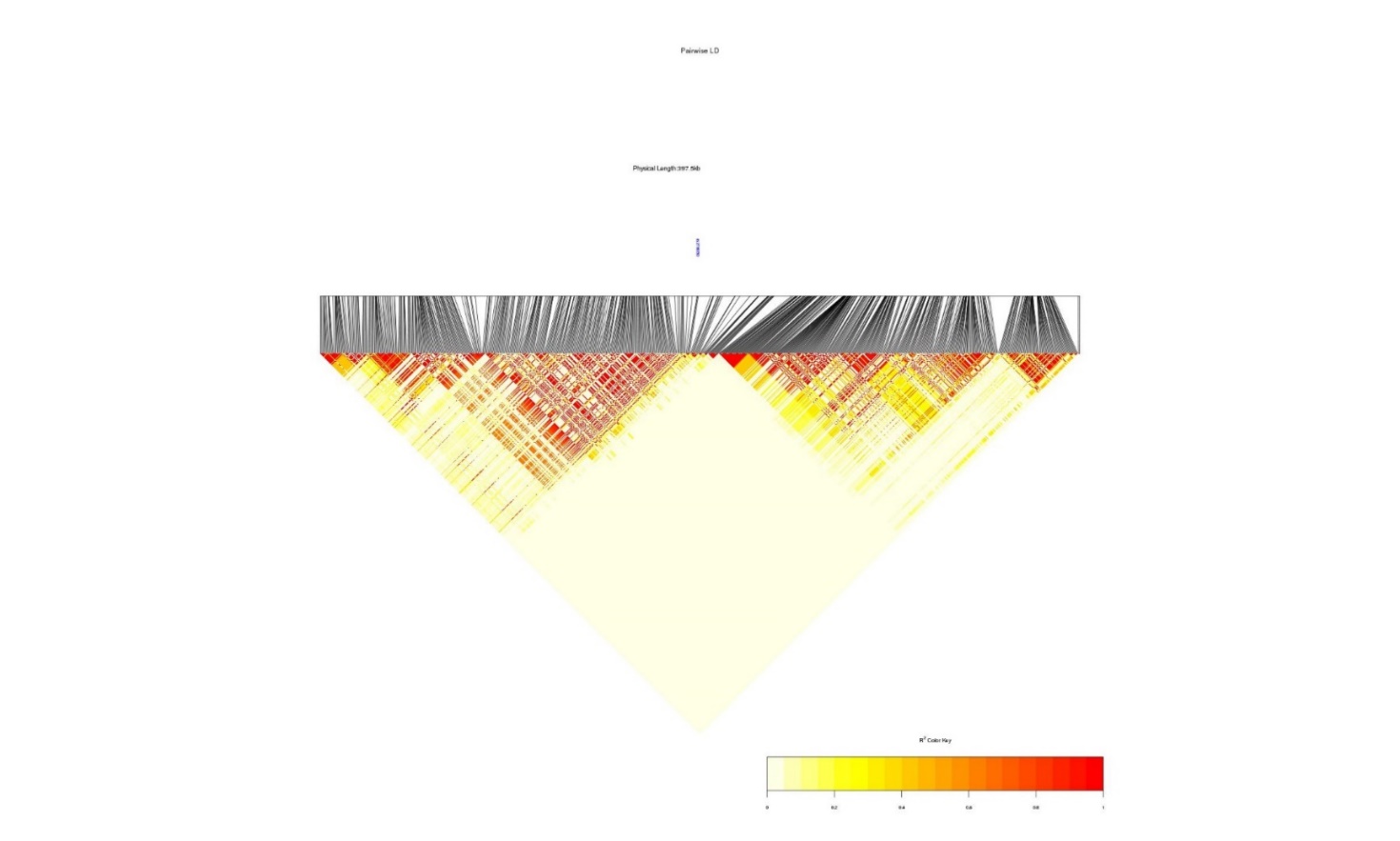

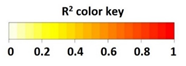


Supplementary Figure S3. Regional association and LD plots of three novel loci

Most significant markers±200kb regions were plotted for (a) *PDE7B* (b) *CDKN2B-AS1* and (c) *UBL3*.

**(a)**  **(b)**


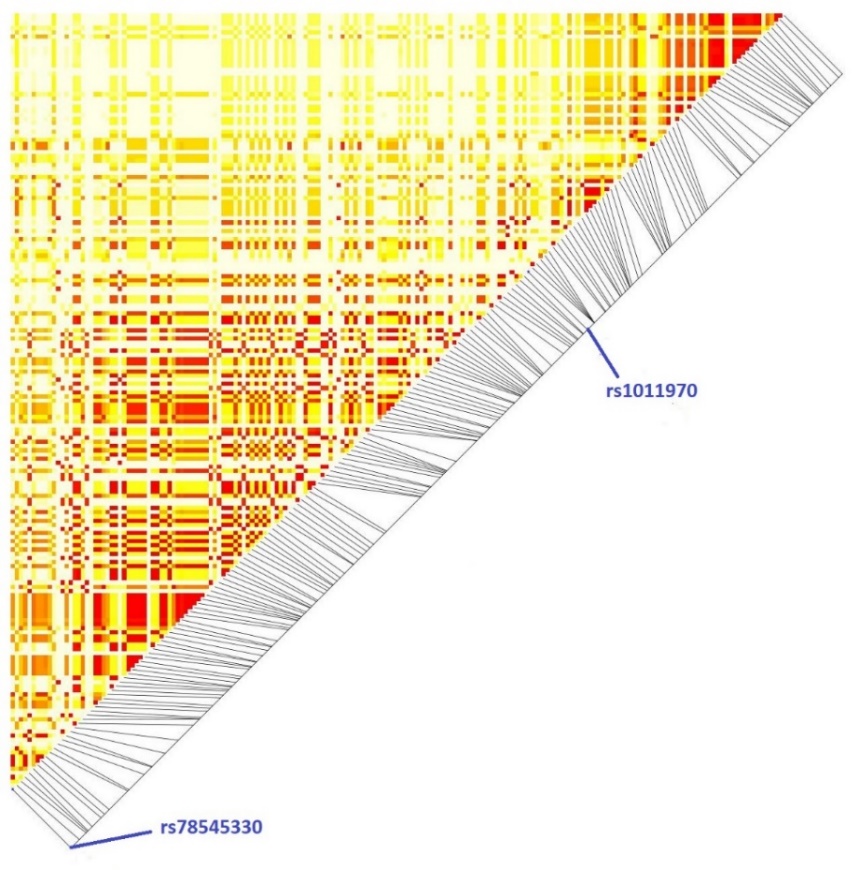

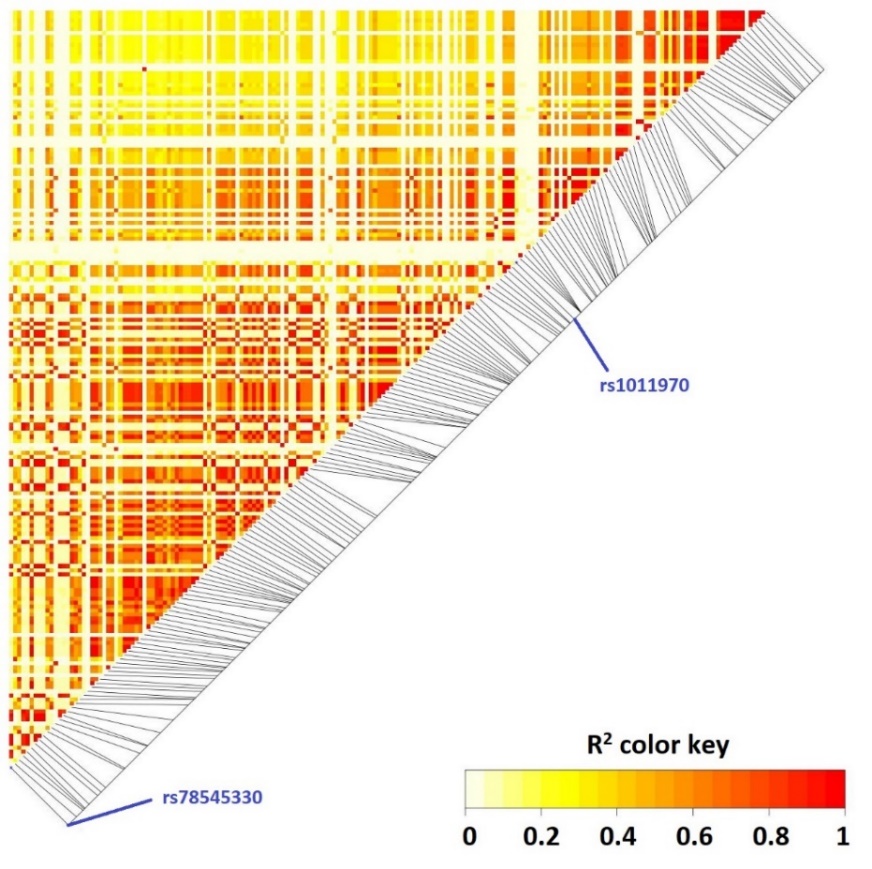


Supplementary Figure S4. LD plots in *CDKN2B-AS1* of two populations

For *CDKN2B-AS1* gene, LD plots were plotted for (a) East Asians and (b) Europeans using data from the 1000 Genomes Project Phase 3. Two labelled SNPs are rs78545330 (the most significant marker in this study) and rs1011970 (previously reported marker from two breast cancer studies of European population). Correlation between these two markers is higher for Europeans than for East Asians.

**References**

1 Han, S. A. *et al.* The Korean Hereditary Breast Cancer (KOHBRA) study: protocols and interim report. *Clinical oncology* **23**, 434-441, doi:10.1016/j.clon.2010.11.007 (2011).

2 Kim, Y., Han, B. G. & KoGES group. Cohort Profile: The Korean Genome and Epidemiology Study (KoGES) Consortium. *International journal of epidemiology*, doi:10.1093/ije/dyv316 (2016).

3 Sung, J. *et al.* Healthy Twin: a twin-family study of Korea--protocols and current status. *Twin research and human genetics : the official journal of the International Society for Twin Studies* **9**, 844-848, doi:10.1375/183242706779462822 (2006).

4 Wen, W. *et al.* Genome-wide association studies in East Asians identify new loci for waist-hip ratio and waist circumference. *Sci Rep* **6**, 17958, doi:10.1038/srep17958 (2016).

5 Breast Cancer Association Consortium. Commonly studied single-nucleotide polymorphisms and breast cancer: results from the Breast Cancer Association Consortium. *Journal of the National Cancer Institute* **98**, 1382-1396, doi:10.1093/jnci/djj374 (2006).
